# *Staphylococcus aureus* Transcriptome Data and Metabolic Modelling Investigate the Interplay of Ser/Thr Kinase PknB, Its Phosphatase Stp, the *glmR/yvcK* Regulon and the *cdaA* Operon for Metabolic Adaptation

**DOI:** 10.3390/microorganisms9102148

**Published:** 2021-10-14

**Authors:** Chunguang Liang, Ana B. Rios-Miguel, Marcel Jarick, Priya Neurgaonkar, Myriam Girard, Patrice François, Jacques Schrenzel, Eslam S. Ibrahim, Knut Ohlsen, Thomas Dandekar

**Affiliations:** 1 Department of Bioinformatics, Biocenter, Am Hubland, University of Würzburg, 97074 Würzburg, Germany; liang@biozentrum.uni-wuerzburg.de (C.L.); anabelen_vadocondes@hotmail.com (A.B.R.-M.); priya.neurgaonkar@uni-wuerzburg.de (P.N.); 2 Department of Environmental Microbiology, Institute of Water and Wetland Research, Radboud University, 6525 AJ Nijmegen, The Netherlands; 3 Institute for Molecular Infection Biology, Josef-Schneider-Straße 2/D15, University of Würzburg, 97080 Würzburg, Germany; marcel.jarick@uni-wuerzburg.de (M.J.); eslam_samir_ragab.ibrahim@uni-wuerzburg.de (E.S.I.); 4 Genomic Research Laboratory, Service of Infectious Diseases, University of Geneva Hospitals, CH-1211 Geneva 14, Switzerland; myriam.girard@genomic.ch (M.G.); patrice.francois@genomic.ch (P.F.); jacques.schrenzel@genomic.ch (J.S.); 5 Department of Microbiology and Immunology, Faculty of Pharmacy, Cairo University, Cairo 11562, Egypt

**Keywords:** metabolism, flux balance analysis, phosphorylation, regulation, riboswitch, PknB, Stp, *yvcK/glmR* operon

## Abstract

Serine/threonine kinase PknB and its corresponding phosphatase Stp are important regulators of many cell functions in the pathogen *S. aureus.* Genome-scale gene expression data of *S. aureus* strain NewHG (sigB^+^) elucidated their effect on physiological functions. Moreover, metabolic modelling from these data inferred metabolic adaptations. We compared wild-type to deletion strains lacking *pknB*, *stp* or both. Ser/Thr phosphorylation of target proteins by PknB switched amino acid catabolism off and gluconeogenesis on to provide the cell with sufficient components. We revealed a significant impact of PknB and Stp on peptidoglycan, nucleotide and aromatic amino acid synthesis, as well as catabolism involving aspartate transaminase. Moreover, pyrimidine synthesis was dramatically impaired by *stp* deletion but only slightly by functional loss of PknB. In double knockouts, higher activity concerned genes involved in peptidoglycan, purine and aromatic amino acid synthesis from glucose but lower activity of pyrimidine synthesis from glucose compared to the wild type. A second transcriptome dataset from *S. aureus* NCTC 8325 (sigB^−^) validated the predictions. For this metabolic adaptation, PknB was found to interact with CdaA and the *yvcK/glmR* regulon. The involved GlmR structure and the GlmS riboswitch were modelled. Furthermore, PknB phosphorylation lowered the expression of many virulence factors, and the study shed light on *S. aureus* infection processes.

## 1. Introduction

Bacteria adapt to different environments to survive changing conditions, and accordingly, they possess an enormous variety of protein kinases involved in signal sensing and transduction. For pathogenic bacteria, infection is ‘growth despite stress’, meaning adaptation to an often-poor metabolic substrate pool the host provides. The most well-described signalling cascades in bacteria are two-component systems (TCSs), which in general consist of a membrane histidine kinase that senses an extracellular signal, autophosphorylates a histidine residue and transfers the phosphate group to an aspartate residue of a response regulator or transcription factor [1]. Ser/Thr phosphorylation, the major regulatory mechanism for cellular functions in eukaryotes, was identified much later also in bacteria [2,3], but it developed fast into an area of great interest due to its involvement in virulence. Ser/Thr kinases have been described in many bacteria, regulating a wide variety of bacterial functions, including glycolysis, protein translation, sporulation and, in pathogenic bacteria, also virulence and antibiotic resistance. Membrane-associated Ser/Thr kinases sense extracellular signals that lead to autophosphorylation and transfer of the phosphate to a serine or threonine residue of a target substrate. The phosphorylation here is not labile, and thus a phosphatase is necessary to remove the phosphate. Less frequently, tyrosine, arginine or cysteine phosphorylation by Ser/Thr kinases has also been described. Unlike TCSs, Ser/Thr phosphorylation integrates a complex signalling pathway in which many biological processes are involved [4,5]. This complicates considerably the study of its role. While the knockout of TCSs often produces a concrete phenotype in the cell, the knockout of a Ser/Thr kinase or phosphatase results in a pleiotropic phenotype in which different pathways are affected.

Recently, a Ser/Thr kinase (PknB, alternatively named Stk or Stk1) and its phosphatase (Stp) were characterised in *S. aureus* [6], a bacterium especially known for its ability to adapt to different environments and its resistance to many antibiotics. *pknB* and *stp* deletion and overexpression strains have been used to study their function in in vitro and in vivo experiments. Here, *S. aureus* phenotypes, such as virulence [7], antibiotic resistance [8], cell wall synthesis [9] and different omics, such as transcriptomics [10], metabolomics [11] and phosphoproteomics data [12], were analysed to decipher the cellular functions that Ser/Thr phosphorylation regulates. The results revealed changes in the virulence, antibiotic susceptibility, cell wall composition and gene expression of *S. aureus*. In *S. aureus* COL and community-acquired MRSA (CA-MRSA) lineage USA300, PknB is a positive regulator of SigB activity for responses to heat and oxidative stress. *pknB* deletion strains have been reported to have higher sensitivity to ß-lactam antibiotics but more resistance to Triton X-100- induced autolysis and to lysostaphin lysis. The reduced SigB activity increases the activity of the quorum-sensing global regulatory *agr*-system resulting in the activation of *agr* RNAII and RNAIII and *hla* (α-hemolysin) virulence effector expression, while *spa* (protein A) is downregulated. Moreover, a *pknB* mutant is more virulent, as tested for a USA300 strain in mice [8].

A number of metabolic enzymes (mainly in glycolysis) are phosphorylated by *S. aureus* PknB, and its deletion affects the expression of genes that regulate central metabolic functions, such as nucleotide biosynthesis, cell wall metabolism and the citrate cycle [6,9,10,12]. According to these different studies and their phenotypic observations, e.g., growth behavior, colony formation and structural and biochemical information, in general the phosphatase Stp is the counter-player of the PknB kinase. Hence, a lot is known about the phenotype of these mutations; however, the metabolic effects of their combined knockout have not been determined yet, nor has anybody looked at the individual enzymes of *S. aureus* primary metabolism and their detailed metabolic adaptation after the individual- or combined-knockout of Δ*pknB* and/or Δ*stp*. Direct metabolomics data on *pknB* mutations are only available on cell wall metabolism [11].

Hence, for our study presented here, genome-scale microarray gene expression data of *S. aureus* NewHG in the late exponential phase were collected. To reveal systematically all pathway changes and obtain a full and far more sensitive view of all involved metabolic changes, we inferred bioinformatically metabolic flux changes from the transcriptome data comparing the wild type and mutations of kinase Δ*pknB*, phosphatase Δ*stp* or both Δ*pknB*Δ*stp* phenotypes. The transcriptome data constrained the inferred fluxes for the full metabolic network and allowed inference on all fluxes calculated, even if there was no significant gene expression change observed for a specific enzyme of the pathway studied. Although this is not a direct metabolite measurement, the prediction error is reduced by fulfilling all network constraints to 5–10% for individual flux predictions (validated in [13] by measuring several metabolites). Further metabolic studies focusing on other carbon sources and growth time points can then build on the results presented here as a pilot study.

Our transcriptome data and the metabolic modelling show that there are many differences in peptidoglycan synthesis, amino acid catabolism and the glycolysis/gluconeogenesis route, while the flux modes calculated allow describing the flux changes for each enzyme of the whole network. To determine how much these mutation effects are (NewHG; *sigB*^+^) or are not (*S. aureus* NCTC 8325; *sigB*^−^) strain dependent or change with different sampling points, we adapted our metabolic model and compared results to *S. aureus* NCTC 8325, for which gene expression data [10] on the key comparison *pknB* mutant versus the wild type are available for the mid-exponential phase. Further, we used interactome data to correlate our results with PknB/Stp in phosphorylation data on cell wall biosynthesising enzymes (Fem proteins) [9]. Further analyses we present shows a clear and strong impact of PknB regulating metabolic adaptation in *S. aureus,* the importance of the *yvcK*/*glmR* regulon and the *cdaA* operon in these processes and delivers after phylogenetic comparisons detailed structure models of the *S. aureus* GlmS protein and GlmR riboswitch.

## 2. Results

**Figure 1:**
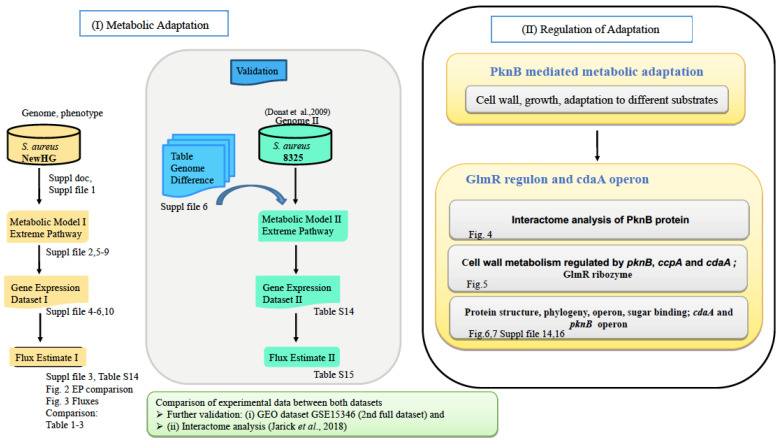
Study workflow of *S. aureus* metabolism. Left: metabolic pathway fluxes were calculated using a first gene expression dataset of *S. aureus* strain NewHG comparing the wild type to *pknB*, *stp* and double-knockout mutants. Validation data (blue boxes) included individual qRT-PCR of selected virulence and metabolic genes and a second gene expression dataset of *S. aureus* strain NCTC 8325 (global comparison). Right: the *glmR*/*yvcK* regulon and the *cdaA* regulon are important for this. Interactions between these regulatory proteins and PknB involve (i) interactome analysis and validation with published datasets and (ii) operon/regulon model as well as phylogenetic analysis (highly conserved phosphorylation sites and data from other Gram-positive bacteria, including alignment, homology model and phylogenetic tree and model of the riboswitch).

The *S. aureus* NewHG phenotype was analysed using the wild-type as well as isogenic mutant strains with deletions of the kinase (*pknB*), phosphatase (*stp*) or both (*pknB* and *stp*). Strain NewHG (also called NewmanHG) is a highly virulent isolate [14], in which the point mutated global virulence regulator *saeS* in strain Newman is repaired [14]. In contrast, strain NCTC 8325 [10] carries a functional mutation of the alternative sigma factor *sigB* due to a mutation in the regulatory *rsbU* gene and a second mutation in the regulatory gene *tcaR* [15]. We chose NewHG as a background strain due to its high virulence properties and its functional *sigB* operon important during stress response. In consequence, we could compare different strain backgrounds, a virulent and a less virulent *S. aureus* strain. First, analysis involved transcriptome analysis of 2414 genes in strain NewHG (Figure 1 left, top; based on the *S. aureus* Newman genome). Genes that were expressed significantly higher or lower are all listed in Table S1, and specific virulence factors are listed in Table S2 (see for both Supplementary Excel File S1). Supplementary File S2 shows the SBML model. Table S3 in Supplementary File S3 compares the enzyme activities of each mutant compared to the wild type, and Table S4 gives the curated NewHG gene expression raw data of the transcriptome measured. All data used in this work, including the Ser/Thr kinase wild type and mutations of kinase (*pknB*), phosphatase (*stp*) and double mutant, have been deposited in the GEO repository (accession no. GSE122362). The reference data GSE15346 used for comparison are also available from GEO [10]. The computational flux model using the software YANAvergence is given with a stochiometric matrix (Table S11 in Supplementary File S8), solution space (Table S12 in Supplementary File S9) and successful convergence for the wild type and mutants (Figure S2). Detailed strain comparisons are given for gene expression data (Tables S5–S7 in Supplementary File S5) and the genome (Tables S8–S10 in Supplementary File S6). In addition, gene identifiers are listed in the supplement (Table S13 in Supplementary File S10). More information regarding *glmR* conservation has been described in the supplement (Supplementary Material).

**Figure 2:**
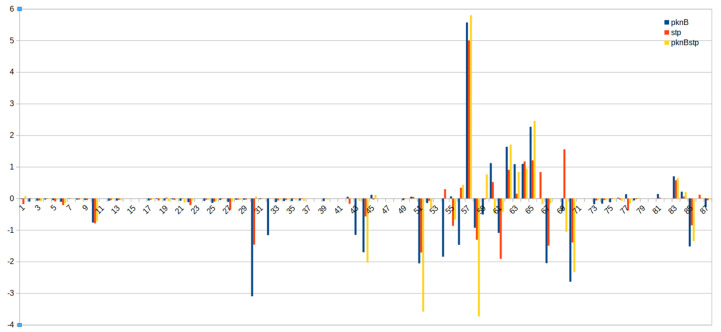
Flux activity changes between Δ*pknB*, Δ*stp*, Δ*pknB*Δ*stp* mutant and wild type. Shown is a compact but data-dense histogram of the flux activity changes. The modes are mathematically calculated, hence there is no statistical deviation. Each of the modes has to be there for metabolic balancing reasons. Some fluxes are small (e.g., fluxes of pathway number 1 to 29). We can detect flux differences down to 5%-10% if they apply to pathways (and not a specific enzyme) [13]. The pathway changes are summarized in Table 1, and a central pathway map overview is given in Figure 3. The x-axis shows pathways according to extreme pathway calculation (as numbered and listed in detail in Table S3 in Supplementary File S3; in Supplementary Material Overview.doc, Table S14 gives a detailed comparison and Table S15 validates the gene expression data by RT-PCR). EPMs contribute to different pathways, but there is no 1:1 correspondence to textbook pathways. Activity changes on the y-axis. Log-fold changes in Δ*pknB* versus WT (blue) of Δ*stp* versus WT (red) and Δ*pknB*Δ*stp* versus WT (yellow) are given in the chart. Values close to 0 indicate no change, and the flux activities remain unchanged in the mutant. The highest flux was observed for EPM 57. It is upregulated in all the three mutants, and this mode is responsible for transaminase activities. The precise cause remains unknown, but the EPM confirms that all the three mutants tend to maintain the pool of glutamate.

**Figure 3:**
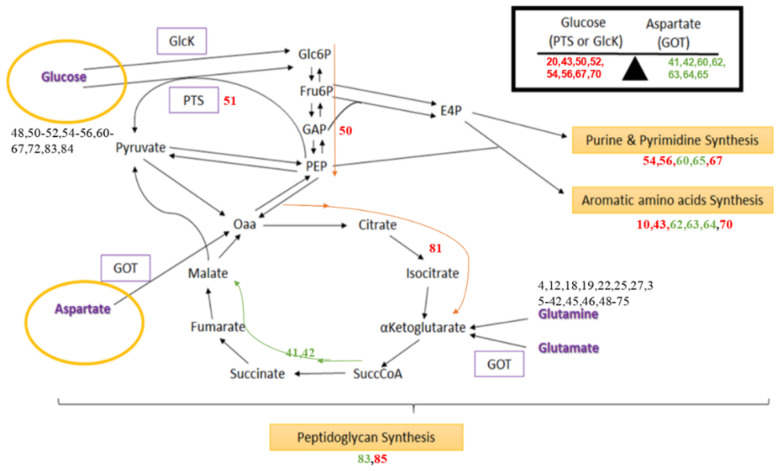
Different metabolic pathways are altered between the WT, Δ*pknB*Δ*stp*, Δ*pknB* and Δ*stp*. The three mutants are compared to the wild type. Shown is the central metabolism and how it is influenced by *pknB*. The corresponding textbook pathways are shown. Fluxes are indicated by arrows. All major (by flux strengths) EPMs that contribute to a specific textbook pathway in NewHG as calculated from the data are shown directly as labels for this textbook pathway. The numbering of the EPMs is given according to Table S3 in Supplementary File S3 (left column; followed by an abbreviated listing of the involved enzymes; next follows the input and output reaction catalysed by the EPM and on the right part the EPM flux activity compared in the wild type and mutants). The colour indicates an up- (green) or downregulated (red) EPM compared to the wild type. The pathways indicated include synthesis of peptidoglycan, nucleotide and aromatic amino acid and catabolism involving GOT.

The collected gene expression data indicate significant deregulated expression of several enzymes in the mutant strains compared to the wild type, but for inference of the metabolic fluxes, the complete enzyme network was meticulously set up. We used pathway information from public databases (KEGG) for *S. aureus* NewHG, and we corrected the list of the enzymes available for NewHG by sequence analysis of the genome sequence and by adding or modifying enzymes as apparent from this analysis (Figure 1 left, middle). Next, the set of pathways available for this enzyme network was calculated using the software YANA (suppl2_SBMLS1.sbml gives the model in SBML/XML format). The software YANAsquare estimates pathway strengths: For this, the directly measured gene expression data (Figure 1 left, bottom) are mapped to the pathways, and gaps or missing pathway information are interpolated for the network. The estimated metabolic flux distribution is then step-by-step adapted to minimise the calculation error using the software YANAvergence. The complete flux distribution for the whole network was calculated. In this way, the pathway fluxes were inferred from the transcriptome data and results are shown in Figure 2 and Figure 3. All elementary modes calculated are given in Table S3 and, major flux changes inferred from the transcriptome data, repeated and found in both experiments, are summarised in Supplementary Material Overview.doc, Table S14. Detailed quality controls in Supplementary Material Figure S2 for wt, Δ*pknB*, Δ*stp* and Δ*pknB*Δ*stp*) indicate the relative changes for the different mutants compared to the wild type. Predictions for expression changes of virulence factors were independently validated by qRT-PCR (see Supplementary Material Overview.doc, Table S15).

To investigate how general our model is with its estimated flux changes, we next analysed the effects of a *pknB* knockout in a second *S. aureus* strain NCTC 8325 versus the wild-type control (Figure 1, middle). We performed similar calculations for this independent transcriptome dataset and different *S. aureus* strains to infer here also metabolic fluxes (genome comparison: Tables S8–S10 in Supplementary File S6; comparative condensed reaction model in Table S11, Supplementary File S8; extreme pathways for NewHG in Table S12, Supplementary File S9; array and gene identifiers in Table S13, Supplementary File S10; strain comparison of fluxes in Table S14 in the Supplementary Material Overview.doc). Note that the metabolic model used was the same for both strains; however, the transcriptome data are strain specific and independent transcriptome datasets for each mutant. For example, different values are presented for EPMs 14–16 comparing NewHG and its pknB mutant. Further independent evidence from other experimental data was considered (e.g., [9]), and protein–protein interaction analysis compared also *pknB* knockout in *S. aureus* NewHG to the wild type. Together, all these data confirm the effect of *pknB* regulation on peptidoglycan structure biosynthesis, potentially with different preferences of carbon use, which helps *S. aureus* to switch between the pathways, depending upon the available nutrient source. Apart from that, we could identify growth conditions and strain-specific differences with metabolic modelling analysis and showed in detail the effects of counter-regulation by Stp and in the combined-knockout strain.

**Figure 4:**
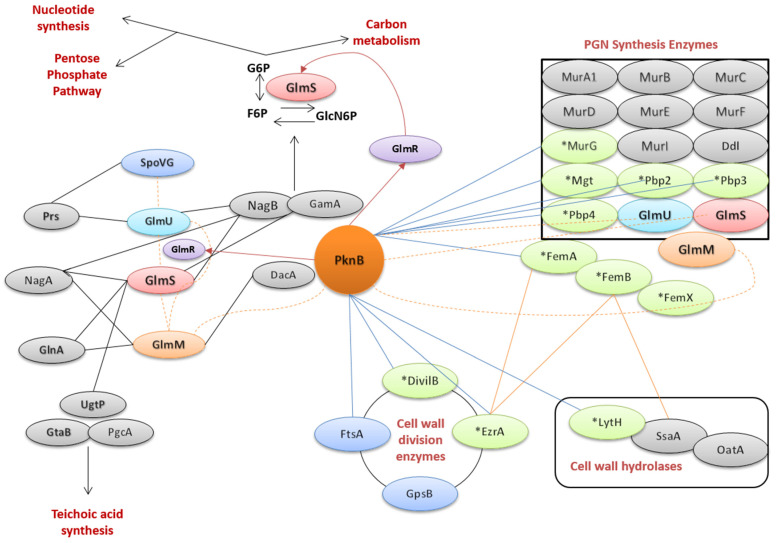
Interactome analysis of PknB protein. The PknB molecule interacts with the proteins involved in central carbon metabolism, nucleic acid synthesis pathways, pentose phosphate pathway, teichoic acid synthesis and the enzymes associated with peptidoglycan (PGN) synthesis. Shown are the different types of interactions: Experimentally verified substrate for phosphorylation by PknB, experimentally verified protein–protein interaction (including data from databases such as the STRING database) and metabolic interactions. Proteins interacting directly with PknB are shown with a blue arrow, and other interactions are indicated by a black arrow. GlmS, GlmU and GlmR proteins are involved in a chain of reactions that regulate central carbon metabolism by interacting with PknB (marked with a maroon arrow). The interactions of Fem proteins (FemA and FemB) are highlighted with an orange arrow. The doted lines indicate different substrates of PknB according to interactome analysis. In the protein interaction network, the central role played by GlmR in association with PknB (green) is shown. GlmR-GlmS-GlmU proteins participate in the cascade of reactions in response to the availability of a nutrient carbon source, thereby regulating different pathways, such as peptidoglycan biosynthesis. The proteins marked with an asterisk (*) are direct interaction partners by PknB substrate phosphorylation according to [11].

**Figure 5:**
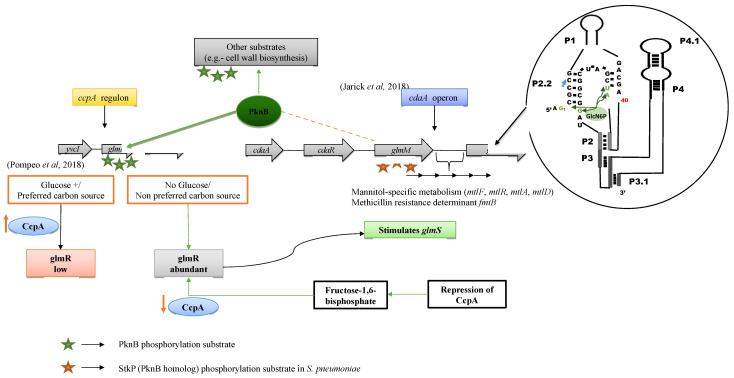
Cell wall metabolism regulated by *pknB*, the *cdaA* operon and the *ccpA* regulon. The different effects in the presence and absence of the preferred carbon source are given. The red stars indicate the direct phosphorylation of GlmM (phosphoproteome analysis and phylogenetic conservation; see [16]). The central molecule PknB (green) regulates GlmR by direct phosphorylation as well as cell wall biosynthesis components (green asterisks). GlmR is a part of the *ccpA* regulon (see [16]), which upon activation by PknB is expressed, which in turn stimulates the expression of GlmS. GlmS is a part of the *cdaA* regulon. The *cdaA* regulon, as shown, has mannitol-specific enzymes and methicillin-resistant determinant FmtB protein conserved in *S. aureus*. These genes can play a vital role in virulence as Gram-positive bacteria have a thick peptidoglycan layer and both these regulons can affect the expression of cell-wall-associated pathways. The different effects in the presence and absence of the preferred carbon source are given. Upper-right circle: secondary structure of the catalytic domain of the *glmS* ribozyme in *S. aureus*. The arrowhead indicates the site of self-cleavage. The guanine at the cleavage site is considered the first residue (G1). Predicted secondary structure of the *S. aureus glmS*–riboswitch aptamer showing the three-junction stem–loop structure typical of the *glmS* riboswitch aptamer. Helices P1, P2, P2.2, P3, P3.1, P4 and P4.1 are highlighted, where P1 to P2 is a catalytic core of the *glmS* riboswitch. Bases interacting with GlcN6P are marked in green. Base-pairing notation is the same as that published previously for *B. subtilis* [17].

**Figure 6:**
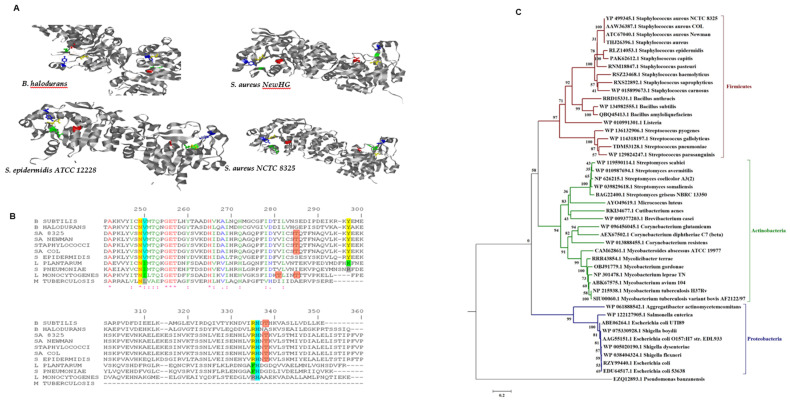
Highly conserved phosphorylation sites in GlmR/Yvck. (**A**) 3D structure analysis of GlmR/YvcK. Crystal structure of *B. halodurans* GlmR/YvcK (PDB ID: 2O2Z), *S. epidermidis* ATCC 12228 (PDB ID: 2PPV), *S. aureus* NewHG and *S. aureus* 8325. The residues of GlmR/YvcK predicted to interact with UDP-Glc (+/−NAc) are highlighted. The figure is visualised with RasMOL software. The structures highlight conserved residues in *S. aureus* shared with *B. halodurans*. Residues in *B. halodurans*: Thr13, Asn217, Tyr264 and Arg300. Residues in S. epidermidis ATCC 12228: Thr14, Asn217, Tyr264 and Arg302. Residues conserved in *S. aureus* NewHG/NCTC 8325: Thr13, Asn216, Tyr263 and Arg301. These residues are predicted to interact with UDP-Glc (+/−NAc). Colour code: Thr (red), Asn (yellow), Tyr (blue) and Arg (green). We predicted here UDP-GlcNAc-binding residues only. NAD-binding residues are shown in MSA. (**B**) Identification of residues involved in UDP sugar binding. Multiple sequence alignment (MSA) of GlmR/YvcK in different bacteria, including several *S. aureus* strains. The highly conserved region of the multiple alignment (residues 240–360) is shown in the figure. Conserved residues are given in yellow. The conserved amino acids highlighted in blue are those found to interact with NAD in the crystal structure. Species and strain names are given on the left. Numbering according to [18]. Boxed in red are conserved threonine sites. A conserved threonine (Thr338 in the MSA) is known by experimental data to be phosphorylated in *B. subtilis* [16] and *L. monocytogenes* [19] and hence predicted by us to be phosphorylated also in *S. aureus* (Thr304 in its sequence, Thr338 in the MSA). A high degree of conservation for the first part of the MSA is indicated in the bottom line by red signs: * (perfect), : (very strong) and . (strong). The complete sequence alignment profile is enclosed in Figure S5. (**C**) Phylogenetic tree of glmR (yvcK). The glmR/yvcK evolutionary history is depicted by using the maximum likelihood method. Different bacterial species are given, and the three clades Firmicutes (blue), Actinobacteria (magenta) and Proteobacteria (green) are distinguished on the right. The percentage of trees in which the associated taxa clustered together is shown next to the branches (boot-strapping values through 1000 iterations). The initial tree for the heuristic search was obtained automatically by applying Neighbor-Join and BioNJ algorithms to a matrix of pairwise distances estimated using a JTT model and then selecting the topology with a superior log-likelihood value. The tree is drawn to scale, with branch lengths measured in the number of substitutions per site. There are apparently three clades in the glmR/yvcK tree.

**Figure 7:**
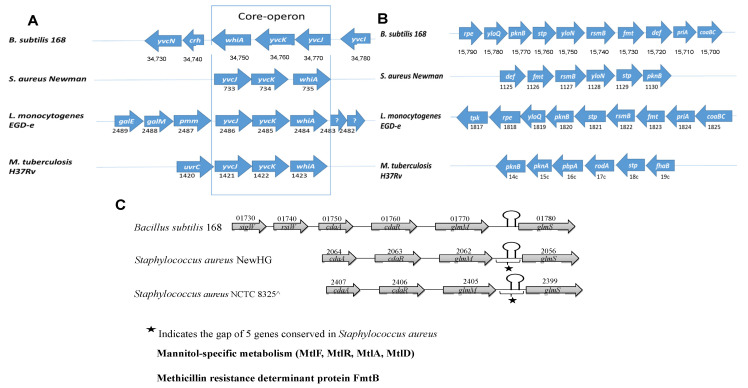
Comparison of regulatory operons in different organisms. (**A**) The glmR/yvcK operon composition in *B. subtilis, S. aureus, L. monocytogenes* and *M. tuberculosis*. Three consecutive genes whiA, yvcK and yvcJ are conserved in all the four organisms, which are core genes. Others are accessory genes located either upstream or downstream of the core gene cassette. Arrows indicate the gene sequence strand, and numbers at the bottom are the locus tag numbers, which are generally the sequential order in the genome. A detailed evaluation of the observed conservation is given in Figure S5. (**B**) The pknB operon composition in *B. subtilis*, *S. aureus*, *L. monocytogenes* and *M. tuberculosis*. Arrows indicate the gene size and the sequence strand, and numbers at the bottom are the locus tag values, which are generally the same as the sequential order in the genome. The genes of the pknB operon appear to be variable in different organisms, *B. subtilis*, *S. aureus* and *L. monocytogenes* are similar in the core composition of the pknB operon, whereas M. tuberculosis is relatively distinct. (**C**) Comparison of the cdaA-cdaR-glmM-glmS operon structure. Compared are the operons from *Bacillus subtilis* 168 and *S. aureus* NewHG and NCTC 8325 (both strains) including the glmS ribozyme. Four mannitol-specific transporters, enzymes (MtlF, MtlR, MtlA, MtlD) and a methicillin-resistance determinant protein (FmtB) are specific for the *S. aureus* cdaA operon, and the genes are located between glmM and glmS. The loop indicates the glmS riboswitch.

The role of the *yvcK*/*glmR* regulon and the *cdaA* operon turned out to be important for this adaptation and was investigated closely (Figure 1, left). The analysis also included the interactions between the regulatory proteins GlmR (Figure 4) and CdaA and PknB involved in metabolic regulation, direct metabolic interaction (interactome analysis; but also known from the classical operon and regulon models; Figure 4 and Figure 5) as well as phosphorylation of GlmR. Figure 5 shows the cell wall metabolism regulated by *pknB*, the *cdaA* operon and the *ccpA* regulon. Ultimately, we found highly conserved phosphorylation sites relying on strain-specific homology models from two GlmR crystal structures (Figure 6A), and based on available sequence data, bioinformatics analysis was performed (Figure 6 and Figure 7). Furthermore, we compared *S. aureus* GlmR-specific data with those of other Gram-positive bacteria by using alignments, homology models and phylogenetic trees of regulatory structures. Moreover, we analysed the structure of the GlmS riboswitch (a self-cleaving ribozyme) in *S. aureus* strain NewHG.

### 2.1. Metabolic Modelling and Pathway Changes for pknB/stp Mutations

In our study, we used transcriptome data to predict metabolic changes by modelling, as there are no direct metabolome data for the different genetic modifications available. We previously established the metabolic modelling method for *S. aureus* under different growth conditions based on gene expression or proteome data [20]. For example, in a *S. aureus* pathway modelling study involving flux estimates for nucleotide and carbohydrate metabolism, the inferred flux predictions from gene expression data were subsequently validated and shown to be correct (+/− 5–10% in flux strength) by direct metabolite measurements of metabolite concentrations [13]. The current study relies on the same method, and we approached the estimate of metabolic flux differences between three mutants with genome-scale gene expression data and minimised the calculated pathway deviations by network analysis. We further validated the metabolic network and flux activity differences found for two mutants (*pknB* and *stp*) compared to the wild type in another *S. aureus* strain with a second dataset. Moreover, the phenotype observations on cell wall metabolism as well as protein–protein interactions directly measured in a recent publication are highly consistent with our calculated results [9]. To derive a metabolic model of *S. aureus* NewHG, first all enzymes of central metabolism according to genome annotation and hand curation were considered. A metabolic model not just looking at the textbook pathways but using all extreme pathways modes (EPM) was calculated from this. One such EPM balances all internal metabolites involved by the combination of its involved enzymes. The EPMs show all pure (“extreme”) pathways accessible for the system and provide hence a generating set to describe all real flux distributions by the combination of the EPMs. EPMs give increasingly detailed information than canonical metabolic pathways. However, they are not a 1:1 transfer of these, but rather EPMs cover often only several enzymes of a pathway. The sum of all EPMs allows considerably more flexibility and better metabolic buffering than just the textbook pathways could suggest. Precisely for this reason, such an analysis is important to better understand the high metabolic adaptation capabilities of NewHG and the detailed effects of *pknB* or *stp* mutation on metabolism. The calculated flux strengths used the information from gene expression data as an approximation for the flux strength. This is possible, as the network and the flux strength calculation even out errors in the individual estimates (similarly all enzymes in the same EPM must be balanced), so low changes in fluxes can also be detected (down to just 5%).

The metabolic model [20] was hence extended to include cell wall metabolism, in particular peptidoglycan synthesis. The different pathways were all given in sufficient detail to investigate the complex changes in enzyme activity according to the gene expression data. Different flux modes were obtained for the metabolic model. The network input file (with the detailed stoichiometric matrix) can be viewed as SBML S1 in SBML/XML format. In total, 149 reactions were taken into consideration, including central carbohydrate, amino acid and lipid metabolism, nucleic acids and peptidoglycan pathways. Next, all balanced metabolic pathways involving this set of enzymes were calculated, which resulted in 87 extreme pathways; reactions and modes are listed in the supplement (Table S3). In particular, the flux balance analysis allows revealing hybrid pathways shared between two or more canonical pathways. We wanted to investigate next the differences in pathway activities under wild-type and mutant conditions. For this, the gene expression data (GSE122362) were mapped on the pathway modes and an optimal fit was calculated using YANAsquare and the fast convergence routine YANAvergence.

Figure 2 visualises the resulting pathway changes and activities for the three mutant strains of NewHG compared to the wild type (for detailed activity values of the entire pathways, see Table S3 in Supplementary Excel File S3). This compact figure is a pathway graph, and higher and lower activities compared to the wild type are shown according to the extreme pathways calculated. On the y-axis, the activity change is calculated, and no change corresponds to the middle position. This is a calculation from flux balance analysis according to the data provided; hence there is no *p*-value given, and the log2 fold-change values (log2FC) are directly calculated according to the network topology. Sensitivity according to metabolite control measurements shows that a 10% change (sometimes even 5%) for a pathway can still be detected by our flux analysis (Cecil et al., 2015) [13]. Table 1 gives an overview on the complex results, and Figure 3 summarises all in a biochemical pathway map (next section). Positive values indicate significant upregulation of this pathway in the corresponding mutant, *pknB*, *stp* or double mutant. The three strains thus do not change the metabolism in the same way, though the effects of *pknB* and the double knockout of the kinase and phosphatase mutants are similar for many pathways. On the x-axis, the number of the extreme pathway modes are listed. Each number represents one extreme pathway from our calculation, usually a modification from a textbook pathway, and the specific enzyme combinations for each pathway for NewHG are listed in Table S3 (Supplementary Excel File S3), and flux activities compared for NewHG and NCTC8325 are shown in Table S14 (Supplemental Material Overview.doc). The pattern shows that some pathways change strongly, while most others change only in the medium-to-moderate level. The biggest negative peak is caused by the regulation in glycolysis and is strongly different. As we can clearly see from Figure 2, most of log2FC values are still close to 0, which indicates these pathway fluxes remain relatively constant in the three mutants compared to the wild type. This observation also implies the rate that is only marginally different by the loss of either PknB or Stp. Instead, the obvious changed modes are 57 (transaminase reactions), 50/51 (upper glycolysis and PTS) and 10/60/63/65/70 (amino acid metabolism).

**Table 1:** Significantly up- and downregulated pathways in *S. auerus* NewHG, *pknB* and *stp* mutants ^1^.

Pathways	Metabolites	Effect on (Fold-Change)			
		Summary	Δ*pknB*/WT	Δ*stp*/WT	Δ*pknB*Δ*stp*/WT
Peptidoglycan synthesis (modes 83, 85)	-	+ GlcK (1.5) −aa	+ GlcK (1.5) −aa (2.8)	+ GlcK (1.5) −aa (1.8)	+ GlcK (1.5) −aa (2.5)
Pyrimidine synthesis (modes 55, 66, 59)	-		NA	+ PTS (1.78) −GlcK (1.8)	−aa −GlcK (1.6)
Purine synthesis (modes 54, 56, 60, 65, 67)	-		+ (2.2)	+ (1.4)	+ PTS (5.5) −aa (13.3)
Nitrogen metabolism: Phe, Tyr, Gly, Glu and Arg metabolism (modes 46, 47, 4, 8, 12, 19, 83, 20, 49, 53, 20, 36, 37, 43, 62, 64, 70, 63, 69, 61)	Phe	+ GlcK −aa	+ GlcK (3.1) −aa (6.2)	+ GlcK (1.9) −aa (2.6)	+ GlcK (3.3) −aa (5)
	Tyr		+ PTS (2.1) −GlcK (2.1)	+ aa (2.9) −GlcK (3.8)	+ PTS (1.7) −aa (2.1)
	Gly	-	-	-	-
	Glu	+ aa (>30)	+ aa (47.5)	+ aa (32)	+ aa (55.5)
	Arg		−Gln (2.2)	NA	NA
Glycolysis (mode 50)	PTS Glc → Pyr	-	−3.3	−1.5	−4.1
	GlcK Glc → aKG	-	−4.2	−3.3	−12

^1^ This table gives the fold-change pathway activity changes, comparing each mutant with the WT as a control. The symbol + means the pathway is upregulated in the mutant and − means the pathway is downregulated. Major carbon sources to fuel pathways examined here are glucose, via the glucokinase (GlcK) or phosphotransferase system (PTS), or aspartate and 2-oxoglutarate via aspartate aminotransferase (AST/GOT). If one looks closer at individual textbook pathways, the picture becomes too complex for this table, as individual enzymes may change differently. Using pathway calculations (elementary modes), we can show that, nevertheless, specific groups of flux modes move and cluster consistently together. GlcK—glucokinase enzyme; PTS—phosphotransferase system; AST—aspartate transaminase (AST/GOT); aa—amino acid. * Glycolysis/Gluconeogenesis is involved in the previous pathways. Detailed pathways and EPM flux modes together with each enzyme and reactions are listed in Supplementary Material Overview.doc, Table S14.

### 2.2. Detailed Analysis of the Pathway Results

After analysing the significantly altered pathways, we created a more detailed list of the metabolic features that changed in the mutants (Table 1; EPM denotes the calculated extreme pathway mode): peptidoglycan synthesis (EPMs 83, 85), nucleotide synthesis (purine and pyrimidine; EPMs 54, 56, 58, 60, 65, 67, 55), aromatic amino acid synthesis (tyrosine, EPMs 61, 63, 69; phenylalanine EPMs 62, 64, 70), amino acid catabolism (threonine, EPMs 10, 11; glutamate, EPM 51; glutamine, EPM 43; aspartate, EPM 57), and pyruvate metabolism (EPM 44) (Table S3 in Supplementary File S3). Table S14 in Supplementary Material Overview.doc provides a detailed list of these pathway changes, enumerating the individual enzyme pathways, comparing mutant *pknB* to the wild type in both strains. These data show that the textbook pathways for primary metabolism, such as glycolysis, pentose phosphate cycle and others, do not dominate the adaptation of *S. aureus*. Moreover, the involved enzymes of one pathway, particularly regarding amino acid metabolism, are not always regulated together up or down at the same time. Instead, metabolic adaptation to PknB-dependent phosphorylation or a lack of phosphorylation causes joined enzyme pathways that are combinations of the well-known textbook pathways of primary metabolism to be up- or downregulated. In general, it was observed that either up- or downregulation of several central pathways is affected by a flux change in concerted metabolites. This is illustrated in Figure 3, which indicates the central metabolism that is influenced by *pknB* and *stp* mutation. The corresponding textbook pathways and all major EPMs that involve enzymes of specific pathways affected by the deletion are shown as up- (green) or downregulated (red) compared to the wild type. In particular, the pathways for peptidoglycan, nucleotide and aromatic amino acid synthesis and catabolism involving aspartate transaminase in the double mutations display higher activity compared to the wild type, while others such as glycolysis are significantly stronger in the wild type. Interestingly, pyrimidine synthesis is dramatically impaired by *stp* and the double mutation, but not by the *pknB* mutation. Since the whole network is not yet completely described, we can only refer to the data we obtained. Indeed, there is a lack of knowledge of which proteins are solely phosphorylated by PknB and dephosphorylated by Stp. Interestingly, phosphoproteome studies show that a number of proteins are still phosphorylated on Ser/Thr residues in a PknB knockout mutant, although PknB is the only known Ser/Thr kinase in the strains used. Moreover, textbook pathways such as pyrimidine synthesis are not one-to-one related to the EPMs. Instead, several EPMs do involve several enzymes of the textbook pyrimidine synthesis and hence contribute, but none of the EPMs covers it completely. For example, regarding the pyrimidine EPMs for the *pknB* mutant, e.g., 55 and 66, are rather simple modes, which agrees with the simple statement on pyrimidine metabolism, i.e., PknB mutation illustrates a similar flux activity in pyrimidine metabolism to the wild type; however, the Stp knockout shows rather individual differences between the modes. EPM 56 is a combination of different pathways, and there are more other enzymes involved (27 enzymes compared to 20 enzymes), so it shows relatively more variation compared to EPMs 55 and 66. Hence, there are clear individual differences for EPMs 55, 56 and 66.

PknB is clearly involved in the regulation of cell wall synthesis but also in numerous other metabolic pathway activities. It switches on, quite specifically, several pathways involving glycolysis (EPMs 50-52), but certain transaminase involving pathways are switched off (EPMs 57, 62–65). Stp, the phosphatase, takes away phosphate groups, mainly from the PknB phosphorylation but also from other proteins (Figure 3, middle). This picture of the Stp phosphatase function is clearly a simplification based on the collected data: Firstly, the metabolic modelling results show that it can only partially antagonise PknB according to our pathway flux activity comparison between different mutants. It is important to observe that this only partial antagonising effect on PknB-regulated pathways suggests that further kinases/phosphatases may be involved (see the Discussion section). The highly complex network leads to fast adaptation to different environmental conditions, and the *pknB/stp* regulatory system is not a Boolean on/off system but rather fine-tunes several pathways. Therefore, we could observe some opposite effects in the kinase and phosphatase mutants. A recent identification of more than 3000 phosphosites localised on Ser and Thr indicates that Ser/Thr kinase signalling, and activity are much higher than previously anticipated [21].

For optimal adaptation of *S. aureus* to changing environmental conditions, there are different routes to produce the same metabolite and, therefore, there are different extreme pathways in which the products are the same. This is the case for peptidoglycan, nucleotide and aromatic amino acid synthesis. Based on the estimated activities, the results reveal that some pathways producing each of these molecules have more activity than the wild type, while others have less. The main difference between these alternative routes is using glucose as a carbon substrate through the glucokinase enzyme (GlcK) or phosphotransferase system (PTS) or not using glucose at all by increasing the amount of aspartate as a substrate through aspartate transaminase (AST/GOT) (Figure 3). However, there is not a clear tendency of specific mutants to activate or inhibit the use of glucose to produce these essential cell components. The observation that pyrimidine synthesis pathways when compared to the wild type are not different in the *pknB* mutant, whereas they are severely impaired in the *stp* mutant, indicates that only *stp* is highly involved in pyrimidine metabolism.

Our observations suggest that the glycolysis pathway from glucose to pyruvate (EPM 44) is less active in the three mutants when compared to the wild type. In contrast, the glutamate synthesis pathway from aspartate and α-ketoglutarate to glutamate and CO_2_ (EPM 57) is the most upregulated pathway in the three mutants. In addition, the reactions from glucose and glutamate to aspartate and α-ketoglutarate (EPM 51) as well as from glutamine and ornithine to arginine and α-ketoglutarate (EPM 43) are downregulated in the two single mutant strains, as well as in the double mutant. Unfortunately, we do not know the exact phosphorylated substrate yet; however, this observation clearly describes the correlation between the glutamate/α-ketoglutarate flux to the mutant strains. Interestingly, two other pathways were affected, comprising just two reactions for transforming threonine into glycine as a common step, followed by the reduction of the coproduct acetaldehyde to ethanol (alcohol dehydrogenase) or oxidation to acetate (aldehyde dehydrogenase). These are EPM 10 and EPM 11 pathways. These amino acid pathways are downregulated in the three mutants and give a clear hint that acetate and glycine are used to synthesise threonine. In Table S3, negative enzyme activity values are displayed for EPM 11. This indicates that the fluxes for this EPM 11 operate in the opposite direction compared to the other EPMs.

In conclusion, the concerted changes allow direct and rapid adaptation to different environmental conditions, several pathways always allow the synthesis of required primary metabolites and enzyme pathways are often jointly reprogrammed by the action of kinase PknB or phosphatase Stp.

### 2.3. Validation of the Inferred Metabolic Responses and Gene Expression Changes in a Second S. aureus Strain

For the above dataset, the flux calculations and gene expression data show that PknB is instrumental in the adaptation to different carbon sources. However, it is not clear whether conclusions regarding the metabolic adaptations that we observed and then translated into flux values can be generalised for *S. aureus*.

To test this, we used data from a knockout strain in the gene *pknB*, compared to the wild type, but considered a different *S. aureus* strain NCTC 8325 as the control. In this case, the samples for gene expression analysis were taken from an earlier time point of the exponential growth phase (GEO dataset GSE15346) [10]. We first tested by detailed genome comparisons between a reference *S. aureus* strain (COL), strain NCTC8325 and Newman, the background strain of NewHG used in this study, whether there are strain-specific differences in the encoded proteins. The metabolic enzymes of primary metabolism are identical among all three strains. There are just two differences from the reference strain COL, looking at NCTC 8325 and NewHG regarding the metabolic enzymes: succinyl-diaminopimelate desuccinylase is absent, and teichoic acid synthesis enzyme SACOL1043, the glycosyltransferase TarM, is specifically present in the COL strain. However, there are a few other protein differences between NCTC 8325 and NewHG that may impact regulation (for strain-specific genes, see Tables S8–S10).

The calculation of the stoichiometric matrix is shown in Table S11 in plain-text format and in SBML format in the file suppl2_SBMLS1. sbml for computation. The resulting extreme pathway modes are given in Table S12. This computational result uses Table S11 as an input file with YANAsquare software and demonstrates there is no difference, as the central metabolic enzymes are the same in NCTC 8325 and NewHG, making the data of GSE15346 [10] an ideal dataset for such a comparison. To achieve a better comparison, the data of the old *S. aureus* NCTC 8325 microarray design (Scienion, Berlin, Germany) were mapped against the identifiers of the new microarray design (Agilent, Palo Alto, CA, USA) in this study, applied in the NewHG study presented here (Table S13). The summarised results for major pathways are shown in Table 2 and are listed in detail in Table S14. Thus, *pknB* mediates amino acid synthesis and strengthens fluxes for this; however, the flux in glycolysis is overall only slightly impaired in both the *pknB* mutation *S. aureus* strains compared (7.1% in NewHG and 6.7% in NCTC 8325; Table 2; details in Table S14). The different elementary modes are listed, as well as the calculated metabolic flux strength in NewHG and in NCTC 8325.

Nevertheless, looking at the inferred pathway differences, the full individual variation between both experiments becomes obvious. Overall, a clear tendency is visible by stronger glycolysis as well as anabolic amino acid metabolism in the NCTC 8325 dataset mediated by PknB. Predominantly, the overall contrasts for central carbohydrate pathways, amino acid metabolism and lipid metabolism are fully supported by this second dataset. A concerted metabolic change in fluxes was observed in both datasets (Table 2). The pathways for nucleotide synthesis, aromatic amino acid synthesis and catabolism involving aspartate transaminase (AST), also known as glutamic oxaloacetic transaminase (GOT), were less active in the *pknB*-knockout strain. There are still some strain-specific adaptations upon *pknB*-knockout mutation, e.g., the pentose phosphate pathway is severely impaired by the absence of PknB in NewHG but not in NCTC 8325. We think that this is due to strain-specific differences. In particular, NCTC 8325 is defective in the alternative sigma factor B and has also other genome differences. However, the exact mechanism for the observed difference in the regulation of the pentose phosphate pathway is not known.

Amino acid metabolism is similarly regulated between NewHG and NCTC 8325; for glycine, there are clear differences. Apart from strain-specific differences in metabolic regulation, some observed differences in gene expression might also be caused by the sampling time point. Though the metabolic activity changes, the central metabolism and amino acid metabolism induced by *pknB* mutation remain highly identical in both *S. aureus* strains.

We also considered further available data, in particular protein–protein interaction data, comparing *pknB* knockout in *S. aureus* NewHG to the wild type. Another impact of PknB is highlighted by this analysis (and supported by the detailed experimental investigation in [9]): PknB promotes peptidoglycan synthesis. This is also observable from the metabolic modelling of the first dataset above, which suggests that the extreme pathway modes for cell wall synthesis are strong in the wild type and impaired in the *pknB*-knockout mutant and could be supported by these data (Table 3) as well as the predicted fluxes from central metabolism contributing directly to cell wall growth.

### 2.4. Involvement of the glmR Regulon and the cdaA Operon

Our gene expression data and the metabolic model show the role of PknB in serine/threonine phosphorylation in amino acid catabolism and the switch between glycolysis (glucose as a substrate) and gluconeogenesis (aspartate as a substrate) to synthesise different cell molecules, such as peptidoglycan, nucleotides and aromatic amino acids. Thus, PknB/Stp modifies the carbon fluxes and cell wall synthesis fluxes.

However, studies from Gram-positive (e.g., *B. subtilis* and *M. tuberculosis*) reveal different regulatory proteins that are involved in this switch. The carbon storage regulator (CsrA), a well-studied protein mainly in Gram-positive bacteria, such as *M. tuberculosis*, regulates the central metabolism (activates glycolysis and inhibits gluconeogenesis) by affecting the stability of mRNAs. CsrA also controls other aspects, such as cell surface properties, motility, quorum sensing, virulence and interactions with animal and plant hosts [22]. Despite these similarities to *pknB*/stp mutant phenotypes, the *csrA* gene does not exist in *S. aureus*. The second candidate is YvcK, a less well-studied protein that used to be labelled in *S. aureus* as a hypothetical protein (NCBI accession no. BAF67006) but was recently identified as GlmR in *B. subtilis* [23]. This protein is essential for bacterial growth under gluconeogenic conditions in *B. subtilis*, *L. monocytogenes* [19] and *M. tuberculosis*. In *B. subtilis*, GlmR is important for the regulation of carbon partitioning between central metabolism and peptidoglycan biosynthesis [23]. In addition, it is also known to be phosphorylated by a serine/threonine kinase in *B. subtilis* [16], *L. monocytogenes* and *M. tuberculosis*. Thus, it represents a promising candidate to bridge the serine/threonine phosphorylation to switching between glycolysis and gluconeogenesis.

The metabolic flux effects (flux data: see supplement; inferred from the gene expression data of *S. aureus*) are best condensed into the following model (Figure 5) on the function of the *glmR*/*yvcK* regulon in *S. aureus* as *glmR* (GlmR transcriptional regulator) and *glmS* (L-glutamine-D-fructose-6-phosphate aminotransferase): central for this operon is a gene cassette comprising *yvcJ* (RNase adaptor), *glmR*/*yvcK* and *whiA* (transcription factor). The alignment profile comprising of *B. subtilis*, *S. aureus*, *L. monocytogenes* and *M. tuberculosis* is shown in Figure 6B and of GlmS ribozyme structure in Figure 5. In addition, a couple of different accessory genes located either upstream or downstream are found in other bacteria around two neighbouring operons: *sigW*-*rsiW* and *cdaA*-*cdaR*-*glmM*-*glmS* in *B. subtilis* [23]. The *whiA*, *yvcK* and *yvcJ* genes are highly conserved in different species, which suggests they are core genes of the *glmR*/*yvcK* regulon that mostly act in a coordinated manner.

The metabolic modelling data suggest that proteins encoded by the *glmR*/*yvcK* regulon and the *cdaA*-*cdaR*-*glmM* operon might be important mediators of the PknB-regulated adaptation (Figure 1, analysis flow) and were investigated more closely. The interactions between these regulatory modules and PknB involve metabolic co-regulation, as well as direct metabolic interaction via the GlmS riboswitch that binds glucosamino-6-phosphate (Figure 5).

### 2.5. Detailed Interaction of PknB with GlmR

An interactome analysis (Figure 4) predicted according to the database (see the Materials and Methods section) that several direct substrates of PknB interact with GlmR [14,24], and GlmR stimulates the activity of GlmS [23], as well as cell wall metabolism proteins. The model is based on known protein–protein interactions, including information from databases such as STRING, experimentally proven interactions between cell wall synthesis enzymes [9] and metabolic interactions (as changing a metabolic pathway). Moreover, we suggest that GlmR may be a direct substrate of PknB, as has been shown in *Streptococcus pyogenes* [25,26]. In *B. subtilis*, the sequence homologue Ser/Thr protein kinase PrkC acts as a substrate for phosphorylation of GlmR, which plays an important role in cell morphogenesis [16]. In *S. aureus*, it has been shown that PknB (also known as Stk1) plays a role in the regulation of cell wall biosynthesis and in drug susceptibility [27]. In *M. tuberculosis*, PknB-mediated phosphorylation with various substrates has been shown [28], and based on sequence, structure and function conservation, we predict GlmR can be indirectly involved in these phosphorylation events in regulating cell shape and cell division. Therefore, we suggest a model, shown in Figure 5, that explains how PknB contributes to cell wall metabolism in the presence and absence of preferred carbon sources. The observed tight metabolic co-regulation (transcriptome data, metabolic modelling; see the previous section) follows from the mutual regulatory interactions between PknB, the GlmR regulon and the *cdaA*-*cdaR*-*glmM* operon (Figure 5). This model is further supported by the genomic organisation of *pknB*, the *cdaA* operon and the *ccpA* regulon. Expression of the *glmS* gene can be stimulated by GlmR under a low-glucose condition or glucose depletion (see [23]; Figure 5, middle). This regulation is governed by the carbon catabolite control protein (CcpA), and GlmR may appear abundant when CcpA activity is low [9,11]. As a result, GlmS is activated, so the system diverts more carbon sources to peptidoglycan biosynthesis (Figure 5, right). However, the regulation may be more complex, since PknB may be capable of phosphorylating GlmR as a secondary regulation pathway, but this is only inferred from our transcriptome data, though supported by published data in other Gram-positive bacteria (Figure 5, left). The resulting effect supports the notion that CcpA may have different isoforms [29]. In addition, as observed by Patel et al. (2018) [23] for *Bacillus subtilis*, the carbon catabolite control protein CcpA represses genes for the use of non-preferred carbon sources when glucose is available, as well as the operon encoding *glmR* (*yvcI-yvcJ-glmR-yvcL-crh-yvcN*). As a result, GlmR should be most abundant when CcpA activity is low. CcpA repressor activity is indirectly stimulated by elevated levels of fructose-1,6-bisphosphate present during growth on preferred carbon sources. During growth on non-preferred, gluconeogenic carbon sources, GlmR will be more abundant, consistent with its role in diverting carbon to PG synthesis under these conditions. Furthermore, the authors proposed a model in which GlmR activates GlmS, and this activity is inhibited when GlmR is bound to the downstream metabolite UDP-GlcNAc. Further supporting experimental observations for the role of the *glmR*/*yvcK* operon in peptidoglycan synthesis are given in Supplementary Material.

### 2.6. Conserved Sequence and Structure of GlmR

Genome sequence analysis revealed similarity in the conserved *glmR* gene cluster in Gram-positive bacteria, including *B. subtilis*, *S. aureus*, *S. epidermidis*, *Streptococcus pneumoniae*, *Lactococcus plantarum* and *Listeria monocytogenes* (Figure 6C). However, GlmR homologous proteins are not restricted to Firmicutes and can also be found in Actinobacteria (e.g., *M. tuberculosis*) and Proteobacteria (e.g., *E. coli*), as shown in the phylogenetic tree of *glmR* (Figure 6C). GlmR possesses probably a conserved UDP-sugar-binding site, as originally described in *B. subtilis* [18], which could also be found in *S. aureus* strains Newman, NCTC 8325 and COL (Figure 6B). These data support that fact that in *S. aureus*, GlmR interacts with NAD, UDP-Glc and UDP-GlcNAc as it does in *B. subtilis*, underlining its potential role in regulating cell wall metabolism. Noteworthy, the deletion of PknB in *S. aureus* affects cell wall metabolism by accumulation of peptidoglycan precursors, including UDP-GlcNAc [9].

Multiple sequence alignment (MSA) further confirmed the high conservation of the putative PknB phosphorylation site of GlmR. GlmR from *B. subtilis* is known to be phosphorylated at Thr-304, as directly measured [16]. In *S. aureus*, Thr-304 could also be the target phosphorylation site, as shown in Figure 6B (in MSA, the counting is different and shifts the threonine to position 338 in MSA).

The predicted ligands of GlmR were further analysed by 3D structure analysis. The GlmR/YvcK structure of *B. halodurans* was directly determined by X-ray crystallography at 2.6-angstrom resolution (PDB ID: 2O2Z) [30]. The the GlmR protein structure of *B. subtilis* was recently modelled and its phosphorylation by PrkC studied [18]. To model the GlmR structure in *S. aureus*, we used the template crystal structure from *S. epidermidis* ATCC 12228 (PDB ID:2PPV; at a high resolution of 2.0 angstroms) and the *B. subtilis* crystal structure as a template and calculated homology models (see the Materials and Methods section) using the strain-specific *S. aureus* Newman, NCTC 8325 and COL sequences (Figure 6A). *S. aureus* and *B. subtilis* were predicted to be homodimers just like the template (high homology found), and the *L. monocytogenes* structure could possibly be a monomer (data not shown) [31]. Figure 6A shows that the identified residues could be involved in binding to UDP sugars, and we verified that these residues are conserved in many Gram-positive bacteria.

### 2.7. General Regulation of the glmR/yvcK Regulon

In addition to GlmR, the biosynthesis of peptidoglycan is tightly regulated by the participation of the *cdaA*-*cdaR*-*glmM*-*glmS* region of the chromosome. This module encodes the major cyclic-di-AMP synthase (CdaA) and a regulator of CdaA, CdaR. GlmS encodes an aminotransferase that catalyses the first reaction of peptidoglycan synthesis. The reaction involves conversion of fructose-6-phosphate (F6P) into glucosamine-6-phosphate (GlcN6P) using glutamine as an amino group donor. Upon stimulation by GlmR, GlmS allows the organism to use a non-preferred carbon source. The switch between the pathways can be regulated by cooperative activity of the *ccpA* regulon and the *cdaA* operon [29]. This involves repression of CcpA, thereby increasing the concentration of GlmR and the ribozyme action of GlmS. The GlmS ribozyme has been shown to be present first in *B. subtilis* [17] and then in *S. aureus* [32]. Including these data, we calculated the conserved secondary structure of the GlmS ribozyme and show it in Figure 5 (top-right corner). GlcN6P has been shown to induce the riboswitch in *S. aureus*. However, the overall rate of the riboswitch is slow in *S. aureus* as compared to other bacteria [32].

The *cdaA* operon centres around the diadenylate cyclase CdaA and controls peptidoglycan biosynthesis in *Lactococcus lactis* [33]. The modulatory effect was reported to come from GlmM. We complement these data here by adding that the whole module (*cdaA*, *cdaR*, *glmM*, mannitol-specific enzymes and *glmS*) is involved in the metabolism of cell wall synthesis in *S. aureus* and tightly interacts metabolically with PknB and GlmR according to our metabolic model and reported protein interactions from the literature and databases (Figure 4). A *glmR*/*yvcK* sequence homology analysis of a broad range of different microorganisms was used to classify the bacteria into the major clades of their phylum, i.e., Firmicutes (clade 1), Proteobacteria (clade 2) and Actinobacteria (clade 3) (Figure 6C). The analysis included the genera *Streptococcus*, *Staphylococcus*, *Bacillus*, *Listeria*, *Streptomyces*, *Salmonella*, *Aggregatibacteria*, *Escherichia*, *Corynebacterium*, *Pseudomonas*, *Nocardia*, *Micrococcus*, *Streptomyces* and *Mycobacterium*. This demonstrates that GlmR/YvcK has evolved into a broad range of bacterial species living in highly diverse habitats. Moreover, they are characterised by different cell wall compositions, shapes and processes of elongation and cell division. Our phylogenetic tree relies directly on the original data, as established by sequencing. For the highest resolution, three *S. aureus* strains were considered, as well as the *S. aureus* consensus sequence. Furthermore, several other staphylococcal species were also considered.

Interestingly, we observed several bacteria lacking *glmR*/*yvcK* in their genomes, such as *Neisseria*, *Haemophilus*, *Helicobacter* and *Chlamydia trachomatis*. This suggests that there are possibly other alternative regulatory mechanisms present at least in some rod-shaped Gram-negative bacteria.

### 2.8. The pknB Operon Is a Regulatory Operon in Many Bacteria

The *pknB* operon involves six genes in *S. aureus*, but the only two genes present in the four microorganisms *S. aureus*, *B. subtilis*, *L. monocytogenes* and *M. tuberculosis* are *pknB* and *stp* (shown in Figure 7B). The protein kinase gene is indicated as *pknB*, and the downstream gene *stp* encodes the corresponding phosphatase. The *pknB* operon from *M. tuberculosis* is considerably different from the other three according to the operon composition. It has unique genes that do not appear in the operons of the three other pathogens: a second Ser/Thr kinase (this is absent from the other microorganisms) and two genes responsible for the rod shape of the bacterium. Genes related to protein translation are present in the other three microorganisms, which have a gene *rpe* encoding ribulose-phosphate 3-epimerase from the pentose phosphate pathway, another for starting DNA replication (*priA*) and one gene for coenzyme A biosynthesis, involved in fatty acid and pyruvate metabolism. In addition, the *L. monocytogenes* operon includes a gene for thiamine diphosphate synthesis, a vitamin B1 derivative that catalyses several reactions of the catabolism of sugars and amino acids (it is present in enzymes such as pyruvate dehydrogenase and decarboxylase, α-ketoglutarate dehydrogenase and transketolase). A detailed view compares the operons from *B. subtilis* 168 and *S. aureus* NewHG and NCTC 8325 (both strains) (Figure 7C). This shows in detail similarities to *B. subtilis*, such as the *glmS* ribozyme, an RNA structure for the glucosamine-6-phosphate riboswitch ribozyme (*glmS* ribozyme) in the 5′untranslated region of the *glmS* gene mRNA, but close by, there is a clear difference of five genes conserved only in *S. aureus*. These data on the impact of the preferred carbon source and *pknB* in directing metabolism towards central metabolism or towards cell wall biosynthesis have major implications for understanding cell wall biosynthesis and methicillin resistance

### 2.9. Virulence Gene Expression and Metabolic Flux Changes Are Tightly Connected in S. aureus

Concerted action of the kinase PknB and its corresponding phosphatase Stp, together with proteins encoded by the *glmR*/*yvcK* regulon, may be important for optimal growth under harsh environmental conditions, e.g., glucose limitation or infection in the host. Besides its impact on metabolic functions, PknB/Stp is also involved in the regulation of virulence factor expression, which might play an important role in infection. Regarding virulence factor expression, we made several interesting observations (Tables S1 and S2 in Supplementary File S1). Many virulence-associated genes are upregulated in the double mutant and *pknB* mutant. In contrast, the *stp* mutant illustrates the effect of downregulation in many virulence genes.

Toxins, including α-hemolysin (*hla*), β-hemolysin (*hlb*), γ-hemolysin components (*hlgA*, *hlgB*, *hlgC*), leucocidin toxin subunits (*lukD*, *lukE*, *lukF*, *lukS*), serine proteases (*splA*-*F*) and cysteine proteases (*sspB*, *sspC*), are upregulated in mutants lacking functional PknB. Thus, the expression of PknB downregulates the transcription of these well-known virulence factors.

In contrast, many virulence factors and their regulators (*agr*, *sae*) appear downregulated in the *stp* mutant compared to the WT. Most strikingly, genes encoding proteases, such as serine proteases (*splB*-*F*), cysteine protease *sspB*, hemolysins (*hlg*, *hla*), leucocidins (*lukD*, *lukE*, *lukF*, *lukS*) and immunomodulatory proteins (*chp*, *scn*, *sbi*), are strongly downregulated in the *stp* deletion strain. As stated above, many of these factors are upregulated in the *pknB* mutant but also in the double-knockout mutant. These results strongly suggest the involvement of serine/threonine phosphorylation in the transcriptional regulation of virulence factors in opposite ways: the action of PknB might downregulate the expression of virulence factors, while Stp-dependent dephosphorylation leads to upregulation. Moreover, several regulators related to virulence factor expression are negatively affected by *pknB* or *stp* deletion, including ArlS (two-component sensor histidine kinase), SaeRS (two-component system response regulator, sensor histidine kinase regulator), Mgr (MarR family regulatory protein) and Sar (staphylococcal accessory regulator T, S, Y and R). This observation is in line with previous findings reporting that phosphorylation of SarA and MgrA modulates virulence and antibiotic resistance in *S. aureus* [34,35].

### 2.10. qRT-PCR Validation of Gene Expression Data

To confirm the microarray results further, qRT-PCR experiments of selected genes were performed. We chose representative virulence genes for qRT-PCR analysis based on the high level of deregulation seen by us in the microarray study. We hence compared the expression of sspB, splB, hla and lip in the wild-type strain NewHG with the pknB and stp mutant strains. In the pknB mutant, expression was upregulated for sspB by 1.7-fold, splB by 1.3-fold, hla by 1.8-fold and lip by 2.1-fold. These data confirm the microarray results (see Supplementary Material Overview.doc, Table S15). Moreover, in the stp mutant, there was corresponding downregulation: sspB by 5.1-fold, sspB 3.9-fold, hla 1.5-fold and lip 2.3-fold.

## 3. Discussion

In this study, we assessed the metabolic phenotypes and the effect of regulation on physiological functions in *S. aureus*, controlled by *pknB*/*stp* during *S. aureus* adaptation to infection. To achieve that, we used direct transcriptome data (strong in identifying direct regulatory effects and adaptations) as well as metabolic modelling based on extreme pathway calculations from these *pknB*/*stp* mutants’ transcriptomics (revealing more subtle changes mirrored in changes in pathways and inferred enzyme fluxes). No direct metabolite measurements were performed. Overall, we observed differences in the glycolysis/gluconeogenesis pathways leading to nucleotide, aromatic amino acid and peptidoglycan synthesis. Based on these results that match with those of previous studies [6,9,10,11], we proposed two regulatory modules that might be interacting with PknB/Stp in *S. aureus*: the *glmR*/*yvcK* regulon and the *cdaA*-*cdaR*-*glmM*-*glmS* module. Furthermore, we analysed the sequence, structure and phosphorylation site conservation of the *glmR*/*yvcK* regulon among different microorganisms and suggested the complex interactome of PknB/Stp, including the previous regulatory modules. Finally, we used transcriptomic data to evaluate the virulence factors controlled by PknB and Stp.

The bioinformatics approach used in this study to infer different metabolic flux activities and to compare different conditions and strains in silico has proven reliable and efficient in former studies [13,15,36]. Nevertheless, validation preferably with direct measurements of metabolites and enzyme activities should be included to predict cellular functions. However, such data are difficult to obtain, and the use of large datasets, e.g., from transcriptomics or proteomics, is valuable to draw reliable conclusions on the role of individual proteins in cellular functions. In general, the actual enzyme activity within the bacterial cell underlies regulation on a transcriptional, translational and post-translational level. Moreover, it can be further modulated by allosteric effectors. However, all these different effects of the inherently complex regulation must be sufficiently balanced for different enzymes acting in a network or pathway context to avoid shortage or accumulation of different metabolites. This network-balancing condition allows inferring metabolic fluxes with reasonable accuracy (about 5–10% for individual enzymes, as calculated in studies with direct metabolite measurements or metabolomics) [13].

Here, we hence modelled the metabolic difference in cellular pathways, as estimated by flux balance computation based on transcriptome data of *S. aureus* wild-type strains and strains lacking the Ser/Thr kinase PknB, the corresponding phosphatase Stp or both proteins. The differences observed in the strains imply that the carbon source diversion may be governed by GlmR/YvcK expression. Furthermore, the data suggest that regulation occurs together with CcpA and PknB under a growth condition of low glucose concentration. To strengthen this hypothesis, we applied a secondary dataset of previously published data from a different *S. aureus* strain (NCTC 8325). The metabolic flux estimation results confirm that the metabolic effects caused by the *pknB* mutation are highly similar also in this strain. Since strain NCTC8325 does not express the alternative sigma factor SigB, we concluded the regulatory role of PknB and GlmR on both carbon source diversion and peptidoglycan synthesis independent of the expression of SigB. However, we noticed that the regulation caused by *stp* mutation shows more differences, which remain to be investigated further. Possibly, other so far unknown phosphatases are also involved in this regulatory network.

Regarding metabolic adaptation mediated by pknB in *S. aureus* and implications for virulence, in the first study using *S. aureus* strain NCTC 8325, several transcriptional changes were observed in a pknB mutant of this strain, which affected genes involved in purine and pyrimidine biosynthesis, cell wall metabolism, autolysis and glutamine synthesis [10]. Most of these pathways were also affected in the present study using strain NewHG, a genetically modified version of strain Newman to obtain a more virulent strain. It shares virulence features with highly virulent clinical isolates (see Herbert et al., 2010 [15]). The inclusion of strains lacking the phosphatase Stp and a strain lacking both activities allowed now a more comprehensive analysis to model growth dependent on the availability of different substrates, which may reflect infection conditions where more specific virulence factors are activated. The metabolic model is made fully available and provides by its extreme pathways (and their flux combinations) all pathways accessible to *S. aureus* for the strain NewHG.

The comparison of each pathway activity between the wild-type strain and the three mutants suggests that Ser/Thr phosphorylation regulates somehow the switch between glycolysis and gluconeogenesis to provide the cell with enough cell wall components, nucleotides and aromatic amino acids. Ser/Thr phosphorylation/dephosporylation in *S. aureus* is complex. There must be other mechanisms active other than Ser/Thr phosphorylation by the Ser/Thr kinase PknB and the Ser kinases HprK and RsbW. Moreover, cross-talk with two-component regulatory systems has been described [21]. This highly complex network leads to fast adaptation to different environmental conditions, and the PknB/Stp system is not an on/off Boolean system but rather fine-tunes several different pathways. Therefore, we could observe clear opposite effects in the kinase and phosphatase mutants, as expected, but also unexpected similar up- or downregulation in all mutant strains. Thus, using flux balance analysis, we see that the flux of EPM57, which is the connection between glutamate to α-ketoglutrate, changes significantly for both the pknB mutant and the stp mutant; however, we do not know the exact phosphorylated substrate yet. In this study, we reported this change and how they are correlated clearly to the mutants. We believe the topic needs to be further investigated in the future.

The altered pathways are also directly related to the catabolism of different amino acids, including aspartate/glutamate, glutamine and threonine catabolism. It is known that *S. aureus* survives through the catabolism of a secondary source and it encodes pathways to catabolise multiple amino acids, including those that generate α-ketoglutarate and oxaloacetate [37]. Moreover, previous studies have proved that preventing the biosynthesis of oxaloacetate in the TCA cycle and its later conversion to phosphoenolpyruvate that is used in gluconeogenesis stops the synthesis of capsule precursors affecting *S. aureus* virulence [38]. In another study, the most virulence-attenuated *S. aureus* mutants identified in a murine model of systemic infections mainly corresponded to defects in metabolism, such as aspartate and pyrimidine biosynthesis and the α-ketoglutarate/malate symporter [39]. Expression of virulence genes, such as hemolysins and proteases, is modulated by PknB expression, as shown in Table S3 in Supplementary File S3. Probably, this is due to rather affecting global regulatory systems, such as the *agr* and *sar* regulators, than direct phosphorylation/dephosphorylation of the virulence proteins. Although both datasets support the main conclusions described above, differences also became clear comparing both experiments and datasets: other EPM activities including secondary metabolites appear relatively different in the comparison of NewHG and NCTC 8325. This may be caused by the different sampling time points or action of the alternative sigma factor SigB, which is not expressed in NCTC 8325. In NCTC 8325, the sample was obtained at the middle exponential phase [10], and in NewHG, it was obtained at the late exponential phase. However, the carbon usage preference governed by PknB remains the same in both strains. It may seem surprising that the carbon use was the same in a comparison of the mid- and late exponential phase. However, we did not compare full medium growth but rather we used a glucose-poor medium (B-medium; see the Materials and Methods section), where the bacteria have to use amino acids to support carbon metabolism [37] *S. aureus* encodes pathways to catabolise multiple amino acids, including those that generate pyruvate, 2-oxoglutarate and oxaloacetate. Obviously, there was no limitation regarding recycling amino acids in the late exponential phase compared to the mid-log phase for these pathways. This is also evident from the unimpaired growth curve (Supplementary Material Overview.doc, Figure S1).

In addition, we applied different levels of constraints for the two strains: there are more enzyme activities derived from the gene expression values of NewHG, than NCTC 8325, due to the older design of the NCTC 8325 microarray (2009 study). Hence, there is no peptidoglycan flux prediction for NCTC 8325 due to a lack of input enzyme activities. However, we have two independent experimental datasets to validate the positive metabolic effects of PknB for peptidoglycan synthesis, and our NewHG microarray data support these flux predictions as well as the interactome data (Table 1, Table 2 and Table 3) [9]. One limitation of the study is that the proposed role of GlmR is based on theoretical predictions from this study and published data from other Gram-positive bacteria. However, phosphorylation of GlmR by PknB in vivo has not been proven yet and remains to be validated in future work.

## 4. Conclusions

Transcriptome data and comprehensive metabolic modelling show that serine/threonine phosphorylation is involved in the virulence capacity of *S. aureus*, as well as regulating its metabolism by allowing growth under a wide range of different substrates. We inferred by bioinformatics a detailed metabolic model for this metabolic adaptation that was quantitatively confirmed by an independent experimental transcriptome dataset from a different *S. aureus* strain. This model suggests that *S. aureus* uses all available metabolic pathways, including combinations of basic pathways to achieve concerted up- or downregulation of biosynthesis and central metabolic pathways. PknB and its intimate connection to the *glmR* regulon and the *cdaA* operon achieve a stable glucose flux, improved aromatic amino acid and nucleotide synthesis and, importantly, peptidoglycan synthesis with cell wall homeostasis, activating central virulence regulators (AgrABCD, ArlS, SaeRS, SarA and MgrA) with implications for virulence and antibiotic resistance. There is no doubt that the activity of kinase PknB versus phosphatase Stp has an important impact on *S. aureus* cell physiology and also virulence. However, the exact role has to be defined depending on growth conditions. Moreover, the characterisation of individual mutants allows predictions of their physiological role. Their regulation is complex (not just antagonistic, sometimes also synergistic) and may differ depending on the activity state of both partners.

## 5. Materials and Methods

### 5.1. Experimental Methods

#### 5.1.1. Generation of Mutant Strains and Phenotype Analysis

The *S. aureus* NewHG mutant strains lacking *pknB*/*stk*, *stp* or both *pknB* and *stp* genes were constructed, as recently described [9].: Briefly, fragments upstream and downstream of *pknB*, *stp* and *pknB*/*stk* were amplified by PCR and cloned together with an erythromycin resistance cassette (*ermB*) into the temperature-sensitive shuttle pBT2. The vector was electroporated into *S. aureus* RN4220, transduced to *S. aureus* NCTC 8325 via phage Φ85 and chromosomally integrated after a temperature shift. Following verification of the correct deletion of the target genes, the gene knockouts were subsequently transduced into strain NewHG via phage Φ85, and deletions were verified by PCR using gene-specific primers.

#### 5.1.2. Media and Growth Conditions for the Bacterial Strains Used in Transcriptome Analysis

We used B-medium, a modified LB medium, which was developed for cultivation of staphylococci [40]. by adding potassium phosphate. As no glucose was added, the bacteria used amino acids for carbon metabolism. *S. aureus* encodes pathways to catabolise multiple amino acids, including those that generate pyruvate, 2-oxoglutarate and oxaloacetate (Halsey et al. 2017 [37]). The authors of this publication found ‘glutamate and amino acids that serve as substrates for glutamate synthesis, particularly proline, function as major carbon sources during growth, whereas other amino acids that generate pyruvate are important for ATP synthesis via substrate-level phosphorylation in the Pta-AckA pathway’. Such a condition would be typical in a low-glucose environment, e.g., in abscesses typically formed by *S. aureus.* Hence, both *S. aureus* strains and all their mutants (*S. aureus* NewHG wt, Δ*pknB*/Δ*stk*, Δ*stp* and Δ*stk*Δ*stp*; *S. aureus* NCTC 8325 and Δ*pknB*/Δ*stk*) were cultivated overnight at 37 °C in B-medium (10 g/L tryptone, 5 g/L yeast extract, 5 g/L NaCl, 1 g/L K_2_HPO_4_) [10] in a shaking incubator at 200 rpm.

The next day, the strains were diluted in 100 mL B-medium (optical density was adjusted at OD600 = 0.05) and again incubated until the cells reached the late exponential growth phase (OD600 = 5.0). Afterwards, RNA was isolated from 7 mL of the bacterial cultures, which were harvested by centrifugation for 10 min at 10,600× *g*, and the bacterial pellet was resuspended in 800 µL of RLT buffer (Qiagen, Hilden, Germany) and mechanically disrupted with glass beads (2 mL Lysing Matrix tubes; MP Biochemicals GmbH, Eschwege, Germany) in Fastprep^®^-24 (MP Biochemicals, Woburn, MA, USA). The cell lysate was centrifuged for 2 min at 18,000× *g*, and the supernatant was used for RNA isolation. RNA was isolated with a RNeasy Mini kit (QIAGEN, Hilden, Germany) according to the instructions of the manufacturer. To remove the DNA template, RNA was treated with RNase-free DNase I (New England, Biolabs^®^ Inc., Frankfurt, Maine, Germany). In contrast to our previous publication, in which we analysed the gene expression of a *pknB* mutant compared to its corresponding wild-type strain NCTC 8325 during mid-logarithmic growth (OD600: 1.0), we now determined the gene expression of Δ*pknB*, Δ*stp* and Δ*stk*Δ*stp* and the wild type in the strain NewHG background.

#### 5.1.3. Microarray Analysis of Transcriptome

The microarray was manufactured by in situ synthesis of 60-base-long oligonucleotide probes (Agilent, Palo Alto, CA, USA), selected as previously described [41]. The array covers >95% of all open reading frames (ORFs) annotated in strains NCTC 8325, UAMS-1 and SA564, as well as Newman, including their respective plasmids. Total RNA was purified from strain NewHG and its mutants grown in B-medium with 10 g of tryptone, 5 g of yeast extract, 5 g of sodium chloride, 1 g of di-potassium hydrogen phosphate and 1 L of aqua dest. to an OD600 of 5, as previously described [10]. For each strain, RNA of three independently grown cultures was analysed. After additional DNase treatment, the absence of remaining DNA traces was confirmed by quantitative PCR (SDS 7700; Applied Biosystems, Framingham, MA, USA) with assays specific for 16S rRNA [42]. Batches of 5 µg of total *S. aureus* RNA were labelled by Cy3-dCTP using SuperScript II (Invitrogen, Basel, Switzerland) following the manufacturer’s instructions. Labelled products were then purified onto QiaQuick columns (Qiagen, Hilden, Germany). Purified genomic DNA from the different sequenced strains used for the design of the microarray was extracted (DNeasy; Qiagen), labelled with Cy5 dCTP using the Klenow fragment of DNA polymerase I (BioPrime; Invitrogen, Carlsbad, CA, USA) and used for the normalisation process [43]. Cy5-labeled DNA (500 ng) and a Cy3-labelled cDNA mixture were diluted in 50 µL of Agilent hybridisation buffer and hybridised at a temperature of 60 °C for 17 h in a dedicated hybridisation oven (Robbins Scientific, Sunnyvale, CA, USA). Slides were washed, dried under a nitrogen flow and scanned (Agilent, Palo Alto, CA, USA) using 100% photon multiplier tube power for both wavelengths. Fluorescence intensities were extracted using Feature Extraction software, version 9 (Agilent). Local background-subtracted signals were corrected for unequal dye incorporation or unequal load of the labelled product. The algorithm consisted of a rank consistency filter and a curve fit using the default locally weighted linear regression (LOWESS) method. Data consisting of three independent biological experiments were expressed as log 10 ratios and analysed using GeneSpring, version 8.0 (Silicon Genetics, Redwood City, CA, USA). A filter was applied to select oligonucleotides mapping ORFs in the Newman genome, yielding approximately 95% coverage. Statistical significance of differentially expressed genes was calculated by analysis of variance [44] using GeneSpring, including the Benjamini and Hochberg false discovery rate correction of 5% (p-value cutoff: 0.05) and an arbitrary cutoff of twofold for expression ratios. Microarray data have been posted on the Gene Expression Omnibus database (http://www.ncbi.nlm.nih.gov/geo; accession nos. GPL10537 for the platform and GSE122362 for the original dataset; see also analysis in Tables S5–S7 in Supplementary File S5).

#### 5.1.4. Quantitative Real-Time PCR

To validate the transcriptome data obtained with DNA microarrays, a qRT-PCR approach was applied. NewHG ∆*stp* and NewHG ∆*pknB* were inoculated from separate overnight cultures (1:200) into 15 mL of fresh B-medium each. We incubated three independent cultures of each strain at 37 °C until the respective OD600 reached 5 ± 0.1, as previously described [10].

Immediately, the bacterial pellets were lysed, and the total RNA was extracted and purified using the RNeasy Mini Kit from Qiagen (Hilden, Germany) according to the manufacturer’s instructions. Using the RapidOut DNA Removal Kit from Thermo Fisher Scientific (Waltham, MA, USA), the DNA was digested from total RNAs. For confirming the absence of DNA traces, qPCRs were performed with assays specific for 16S rRNA.

cDNA synthesis was performed with SuperScript IV from Thermo Fisher Scientific and random primers. We used the SsoAdvanced Universal SYBR Green Supermix (BioRad, Feldkirchen, Germany) for qRT-PCR according to the manufacturer’s instructions. Five sets of primers (Table 4) were designed for the optimal annealing temperature and specificity and tested for efficiencies (all in the range between 90% and 110%) to quantify four genes and the housekeeping gene. For each strain, 3 independent cDNAs were generated from 3 independent cultures. Relative abundances were calculated using *gyrB* as a housekeeping gene with the method of Pfaffl [24] to correct for primers efficiencies. Non-template controls verified the absence of any contaminating templates.

### 5.2. Bioinformatics Methods

#### 5.2.1. Transcriptome, Proteome, and Protein Interaction Data Retrieval

Transcriptome data of *S. aureus* analysis were generated, as described above, and retrieved from the NCBI Gene Expression Omnibus (GEO) dataset (GEO accession no. GSE15346) [10].

Proteome data and inferred metabolic adaptations by flux modelling of changes after diauxic shift [20] were used for comparison of the predicted metabolic changes to this different condition.

Protein interaction data were generated before [9], as described briefly in the experimental section, and used for interaction prediction validation. The new transcriptome data on the NewHG strain from the experiments for transcriptome analysis are given in Table S4.

#### 5.2.2. Metabolic Model Reconstruction and Extension

*S. aureus* metabolic network models were previously reconstructed using annotations of KEGG and TIGR [20]. The first bioinformatics model was established for *S. aureus* COL, as it is one of the most referenced and investigated strains. The second network model was based on *S. aureus* NewHG [14], a highly virulent strain. This second metabolic model was achieved by adaptation of the COL metabolic model, and the enzymes were present if they could be mapped by the ortholog gene pairs. The missing enzymes from KEGG were added to close the gaps after careful re-annotations and comparisons, considering all available evidence, including biochemical analysis proof, potential moonlighting catalytic domains and other published observations. Here, the peptidoglycan synthesis pathway was added to the *S. aureus* COL model developed previously, as mentioned before [20]. The reactions comprising the whole pathway were grouped into 5 sets to keep the model as condensed as possible (Table 1).

#### 5.2.3. Extreme Pathway Computation Using YANAsquare

Extreme pathways are defined as a unique, minimal set of enzymes and enzyme complexes (participating reactions) to support the steady-state operation of a metabolic network with irreversible reactions to proceed in appropriate directions (that balances all involved internal metabolites). Together these extreme pathways limit the solution space of metabolic possibilities for the metabolism of the organism modelled [45]. These pure (extreme) pathways combine in vivo to provide their joint metabolic fluxes in all metabolic situations. This includes the well-known textbook pathways, such as glycolysis. In the results, all the extreme pathways that provide major contributions to one of these textbook pathways are given as contributing to that textbook pathway (the initial figures in results show this also in detail).

Extreme pathway analysis was performed using YANAsquare software [45]. To calculate the relative flux through all the necessary reactions involved in the biochemical reaction regulated by Ser/Thr phosphorylation, information from previous metabolic models was considered, too. After applying YANAsquare software to this new *S. aureus* extended model, a list of extreme pathways and their mathematical representations (the null space) were obtained for further analysis.

#### 5.2.4. Pathway Activity Estimation and Comparison Using YANAvergence

To calculate each pathway activity based on expression data of the WT and mutants, the YANAvergence routine protocol was applied [20]. This is a convergence routine that minimises the difference between experimental data and calculations. It iterates until it cannot significantly improve the convergence from the previous iterations. The R script was run with different transcriptomic data each time, so one activity per extreme pathway and WT or mutant was obtained. After that, the mutant activities of each pathway were compared individually to WT activity using the ratios Δ*pknB*/WT, Δ*stp*/WT and Δ*pknB*Δ*stp*/WT. Transcriptional data were measured, and the statistical analysis is given accurately in the GEO data (accession nos. GPL10537 for the platform and GSE122362). However, regarding the metabolic flux inference, these results are mathematical flux calculations. There is no statistical error, and it is a mathematical calculation. We tested the accuracy of flux calculations from gene expression data measuring directly metabolite concentrations. We found the limit of sensitivity is at least a 10% difference, as proven by direct metabolite measurements (Cecil et al., 2015 [13]).

Each pathway is a subset of enzymes or reactions that transform specific substrates into products with no accumulation of internal metabolites. The activity associated with each pathway is based on the best fit of transcriptomic data, and it was used in this study to discover the differences in metabolic fluxes between the WT and three mutants of *S. aureus*. Zeroes as entries indicated no known fluxes or flux not detectable; 50 was the starting value, and the maximum value was 100. Convergence criteria were successfully met for all the simulations with different numbers, i.e., result values are as follows: for the WT: iter 310, final value 55.847131; for Δ*pknB*: iter 280, final value 55.141834; for Δ*stp*: iter 300, final value 53.315411; and for Δ*pknB*Δ*stp*: iter 330, final value 55.627214 (“iter” denotes the iteration number in the optimisation using YANAvergence; the final value for this study is the sum of absolute values of the deviations instead of the standard deviation). Convergence is achieved for values below 100, as validated by metabolite measurements [13]. The convergences are further examined and plotted in Supplementary Material (Figure S2).

#### 5.2.5. Protein Interaction Network Analysis

The *S. aureus* protein–protein interactions (PPIs) were studied using the STRING database tool, version 9.1 [46]. This approach was used to take into consideration the broad collection of datasets, including text mining and homology determination, adding together with experimentally validated PPIs. Finally, all available data regarding interaction were analysed.

The interaction network of Stk, Stp and FemX/A/B proteins among cell wall synthesis and cell division proteins relied on experimental data by bacterial two-hybrid analysis [9]. Phosphorylation was also determined [9]: Stk phosphorylated FemX but did not phosphorylate FemA or FemB in the in vitro kinase assay. Stp dephosphorylated Stk-dependent phosphopeptides of FemX.

#### 5.2.6. Comparative Sequence Analysis and Phylogenetic Analysis

The protein sequence BLAST was performed online using the default parameters and certain species or phyla desired to look for *yvcK*/*glmR* homologues. Multiple sequence alignment (MSA) was then performed using MUSCLE [47], and resulted profiles were analysed with MEGAX [48]. The evolutionary history was inferred by using the maximum likelihood method based on the JTT matrix-based model. The initial tree for the heuristic search was obtained automatically by applying Neighbor-Join and BioNJ algorithms to a matrix of pairwise distances estimated using a JTT model and then selecting the topology with a superior log-likelihood value. The phylogenetic analysis involved 42 putative YvcK/GlmR protein sequences. The bootstrap test was carried out with 1000 iterations using the highly conserved region of the YvcK/GlmR protein.

#### 5.2.7. RNA Folding

The sequence-based identification of the metabolite-dependent *glmS* element was predicted using the Riboswitch finder (http://riboswitch.bioapps.biozentrum.uni-wuerzburg.de) accessed on July, 2020 and the RNAfold web server (http://rna.tbi.univie.ac.at) based on the latest ViennaRNA package (version 2.4.11).

#### 5.2.8. Phylogenetic Analysis of GlmR

Because *glmR*/*yvcK* of *B. subtilis* is involved in survival under different gluconeogenic conditions, it might be more similar between microorganisms that share specific niches, and thus, they are adapted to use the same substrates for energy production and cell components synthesis. For that reason, a phylogenetic tree of different bacterial species was created with MEGAX using the maximum likelihood method based on the sequence homology of the GlmR/YvcK protein.

#### 5.2.9. 3D Structure Prediction Using Swiss-Model

To validate the residues of binding sites, 3D structure prediction was performed using the online platform SWISS-MODEL [49]. Relative protein sequence alignment was profiled to determine the closest related template, and the coordinate data were obtained from the PDB. Using a linear function, the server calculates a score that represents the likelihood of each atom to bind a ligand atom. In the case of *S. aureus*, an attempt was made to build a 3D structure model using available sequences of the proteins and by structurally comparing it with alternative bacterial templates and the binding residues.5.

## Figures and Tables

**Table 2:** Metabolic pathway effected by *pknB* mutation in *S. aureus* NCTC 8325 [10] and flux activity comparison with this study (*S. aureus* NewHG).

Pathway	EPM ^1^	NewHG ^2^	NCTC 8325 ^3^
Glycolysis	50	−7.1%	−6.7%
Pentose phosphate pathway	51	−72.7%	+7.6%
TCA (cit)	81	−4.4%	−4.8%
TCA (succ)	41, 42	+21.2%	−1.9%
Purine metabolism	60, 65	+10.2%	−20.8%
Purine metabolism	54, 56	−21.4%	−17.3%
Purine metabolism	67	−10.2%	+27%
Amino acid (Phe, Tyr)	62, 63, 64	+313%	+17.6%
Amino acid (Phe, Tyr)	70	−96.9%	−21.8%
Amino acid (Gly)	10	−41.1%	63.2%
Amino acid (Arg)	43	−0.7%	−100%

^1^ EPM—extreme pathway mode (applies to both strains); ^2^ NewHG—*S. aureus* Newman strain derivation with allele saeS^L^; ^3^ NCTC 8325 standard laboratory strain. A positive value indicates a gene upregulated in the *pknB* mutant, whereas a negative value means a gene downregulated in percentage. Compared to the wild type of NewHG and NCTC 8325, the impact of *pknB* mutation on central metabolism; purine, pyrimidine and partial amino acid synthesis; and metabolism (Phe, Tyr, Gly, Arg) is fully supported and validated by this second dataset. However, there are clear strain-specific differences in less central pathways, and detailed flux activity differences are compared in Table S14 (Supplemental Material Overview.doc). Flux changes down to 5–10% are reliably detected by flux calculations, as validated by direct metabolite measurements [13].

**Table 3:** *S. aureus* NewHG metabolic changes under *pknB*/*stp* mutation.

Pathway	∆*stp*	∆*pknB*	∆*stp*∆*pknB*
Pyrimidine synthesis (modes 55)	45.1% Impaired (−)		37% Lower activity as compared to WT (−)
Purine synthesis (modes 60, 65)			Higher activity (+)
Aspartate, α-ketogluarate, glutamate (mode 57)	+	+	+
Glutamate synthesis	+	+	+
Peptidoglycan synthesis (mode 83)	+	+	+
Peptidoglycan synthesis (mode 85)	−	−	−
* Cell wall morphology and thickness ^1^			
* Logarithmic phase	* Larger (7%) Thicker (26%)	* Larger (8%) Thinner (23%)	* Larger (16%)
* Stationary phase	* Smaller (4%) Thicker (38%)	* Larger (15%)	* Larger (15%)

^1^ Data from [9]. Comparison of the flux changes in the different mutants versus the wild type. Note that there is also a strong flux into peptidoglycan synthesis (modes 83, 85) collecting the synthesis reactions above into cell wall components. * Experimentally determined [9].

**Table 4:** Primer sequences for qRT-PCR.

Gene	Name	Nucleotide Sequence (5′ to 3′)
16s rRNA Control	16rRNAup	TTGCTTCTCTGATGTTAGCG
	16rRNAdown	TCTAATCCTGTTTGATCCCC
splB	SplB1	GCGTGCAATAGAACGTGGACC
	splB2	GCTCACCAGCTTTAGCCCCT
sspB	SspB1	TGGCAGTTGTTGGGAACGCT
	SspB2	ATGGTCGCCATTGGATACTGG
hla	Hla1	CGAAAGGTACCATTGCTGGT
	Hla2	GGTAGTTGCAACTGTACC
lip	Lip1	TGGTGGACAAGCACAAGCAG
	Lip2	GTTGCTGTTCGTCAACACCG
gyrB	GyrB1	GGTGGTTTACATGGTGTTGG
	GyrB2	CCTGTGTTATCAGTTGTGCC

## Data Availability

All data are available from the paper and its supplementary materials. Transcriptome data have been deposited with GSE122362, software YANA is available at https://www.biozentrum.uni-wuerzburg.de/bioinfo/computing/yanasquare/ and https://www.biozentrum.uni-wuerzburg.de/bioinfo/computing/yanavergence/.

## Supplementary Materials

The following files and data are available online at https://www.mdpi.com/article/10.3390/microorganisms9102148/s1. A doc file provides the Supplementary Material overview. It contains Figure S1: Growth curve of *S. aureus* strains NewHG WT and its isogenic pknB, stp and pknB/stp mutant strains. Figure S2: Convergence plots of (A) WT, (B) *pknB*, (C) *stp* and (D) *pknBstp*. Conservation of *glmR* including Figure S3: The complete sequence alignment profile: identification of residues involved in UDP sugar binding. Table S14. Flux activities comparing WT and *PknB* mutant in *S.aureus* strains NewHG and NCTC8325. Table S15: qRT-PCR confirmation of microarray data. Moreover, we provide 10 Supplementary Files that are not Word files (most are in Excel): Supplementary File S1 contains Table S1 (Genes whose transcription was significantly higher or lower expressed) and Table S2 (Expression of virulence factors). Supplementary File S2 shows the SBML metabolic model. Supplementary File S3 has Table S3 (Enzyme activities of each mutant compared to the WT). Supplementary File S4 has Table S4 (NewHG gene expression raw data). Supplementary File S5 contains Table S5 (Gene expression of WT versus double mutant), Table S6 (Gene expression of WT versus *pknB* knockout) and Table S7 (Gene expression of WT versus *stp* knockout). Supplementary File S6 contains three tables on genome annotation: Table S8 (Genomic comparison between *S. aureus* NCTC 8325 and Newman), Table S9 (Genomic comparison between *S. aureus* NCTC 8325 and COL) and Table S10 (Genomic comparison between *S. aureus* NCTC 8325 and COL). Supplementary File S7. Stochiometric matrix and reactions involved in the condensed model of *S. aureus* NewHG—model file in cvs format. Supplementary 8 contains Table S11 (Stochiometric matrix and reactions involved in the condensed model of *S. aureus* NewHG)—model file in Excel format. Supplementary 9 contains Table S12 (Extreme pathway modes of *S. aureus* NewHG)—solution space. Supplementary File S10 contains Table S13 (Gene identifiers of two microarray design mapping and comparing NewHG-Agilen with NCTC 8325-Scienion; gene symbols are also added).

## Abbreviations

TCS	Two-component system
PknB (Stk or Stk1)	Ser/Thr kinase
Stp	Phosphatase of PknB
CA-MRSA	Community acquired methicillin-resistant Staphylococcus aureus
qPCR	Quantitative polymerase chain reaction
cDNA	Complementary deoxyribonucleic acid
GEO	Gene Expression Omnibus
ATCC^®^	American Type Culture Collection
GlcK	Glucokinase enzyme
PTS	Phosphotransferase system
AST/GOT	Aspartate transaminase
MSA	Multiple sequence alignment
NAD	Nicotinamide adenine dinucleotide
UDP-Glc	Uridine diphosphate glucose
UDP-GlcNAc	Uridine diphosphate N-acetylglucosamine
GlcN6P	Glucosamine-6-phosphate
PPI	Protein–protein interaction
