# Supplementary material for: Staphylococcus aureus Transcriptome Data and Metabolic Modelling Investigate the Interplay of Ser/Thr Kinase PknB, Its Phosphatase Stp, the glmR/yvcK Regulon and the cdaA Operon for Metabolic Adaptation
Source: Microorganisms. 2021 Oct 14;9(10):2148. doi: 10.3390/microorganisms9102148 (PMC8537086; doi:10.3390/microorganisms9102148)
Supplement: Supplementary file 1 [file microorganisms-09-02148-s001.zip › Supplementary_Material_Overview.pdf]

Supplementary Materials

Supplementary Materials: The following files and data are available online at www.mdpi.com/xxx/s1:

This doc file provides a supplementary material overview and contains:

-**Overall investigation** and **Figure S1**: Growth curve of *S. aureus* strains NewHG wt compared to its isogenic *pknB*, *stp* and *pknB/stp* mutant strains.

- **Figure S2**: Convergence plots of (A) WT, (B) *pknB*, (C) *stp* and (D) *pknBstp*.

-Additional data on **conservation of *glmR*** including **Figure S3**: Identification of residues involved in UDP-sugar binding in GlmR/YvcK protein (complete sequence alignment profile of GlmR/YvcK in different bacteria including several S. aureus strains).

-**Table S14**. Flux activities comparing WT and *PknB* mutant in *S.aureus* strains NewHG and NCTC8325.

-Table S15: qRT-PCR confirmation of microarray data

We provide the other supplementary files **Table S1-S13** not in this doc File. Apart from Suppl2 (model file in SBML format) all Supplemental files are in excel format.

Suppl1 contains **Table S1** (Genes whose transcription were significantly higher or lower expressed) and **Table S2** (Expression of Virulence factors).

Suppl2 shows the **metabolic model** in **SBML format**.

Suppl3 has **Table S3** - Enzyme activities of each mutant compared to the WT.

Suppl4 has **Table S4**, NewHG gene expression raw data.

Suppl5 contains **Table S5** (Gene expression of WT versus Double Mutant), **Table S6** (Gene expression of WT versus *pknB* knockout) and **Table S7** (Gene expression of WT versus *stp* knockout).

Suppl6 contains three tables on genome annotation regarding the specific genes for each strain: **Table S8** (Genomic comparison between *S. aureus* NCTC 8325 and Newman), **Table S9** (Genomic comparison between *S. aureus* NCTC 8325 and COL) and **Table S10** (Genomic comparison between *S. aureus* NCTC 8325 and COL).

Suppl7 Stochiometric matrix and reactions involved in the condensed model of *S. aureus* NewHG – model file in cvs format-

Suppl8-**Table S11**. Stochiometric matrix and reactions involved in the condensed model of *S. aureus* NewHG – model file in excel format.

Suppl9-**Table S12**. Extreme pathway modes of *S. aureus* NewHG – solution space.

Suppl10- **Table S13**. Gene identifiers of two microarray designs mapping and comparing NewHG-Agilent with NCTC 8325- Scienion, gene symbols are also added.

The overall aim of our investigations is to understand the effect of the Ser/Thr kinase *pknB*, its phosphatase *stp* and to understand antagonistic or synergistic by studying the wildtype and double knockout. The workflow focuses first on calculation of metabolic fluxes from transcriptome dataset on *S. aureus* strain NewHG and comparing it to *pknB*, *stp* and double knockout. Secondly, we validated the results by individual qRT-PCR of selected virulence and metabolic genes further we performed the global comparison with *S. aureus* strain NCTC 8325 (Figure 1). The phenotypic data was also taken into consideration to explore effects on cell wall metabolism and its correlation with the phosphorylation mechanism.

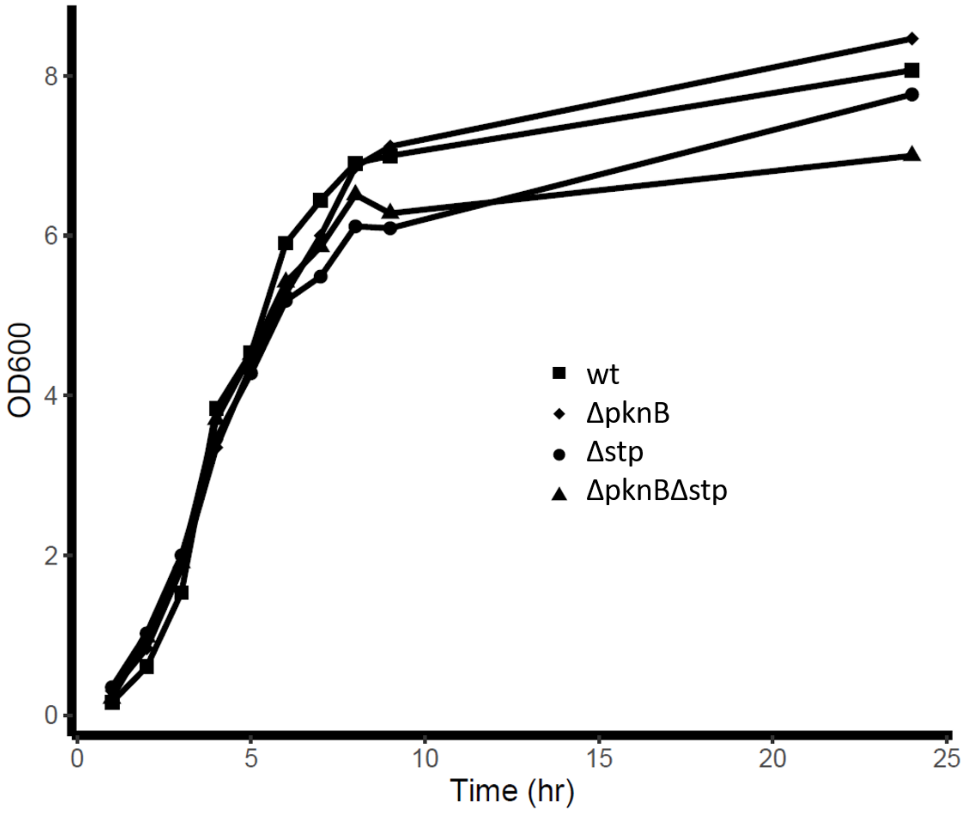

Figure S1: Growth curve of *S. aureus* strains NewHG wt compared to its isogenic pknB, stp and pknB/stp mutant strains. Growth in B-medium over a period of 24 h is shown. The generation time of *S.aureus* strain NewHG is 46 min under the applied conditions. In fact, we even measured the growth curves for wildtype and all three mutants and there were no striking differences until all strains reached an OD600 of approximately 5. Later in growth phase the double mutant and the *stp* mutant showed reduced growth rates. The figure compares the growth behavior of the mutants with the wild type strain using a medium mimicing conditions in an abscess (low glucose) using B-medium. The growth happens well for *S. aureus* in this specific medium also up to and beyond OD 5.0. Beyond it, the exponential growth stops and becomes more steady state. There are no strong differences in the growth behavior, however, the double mutant clearly achieves less as maximum growth.

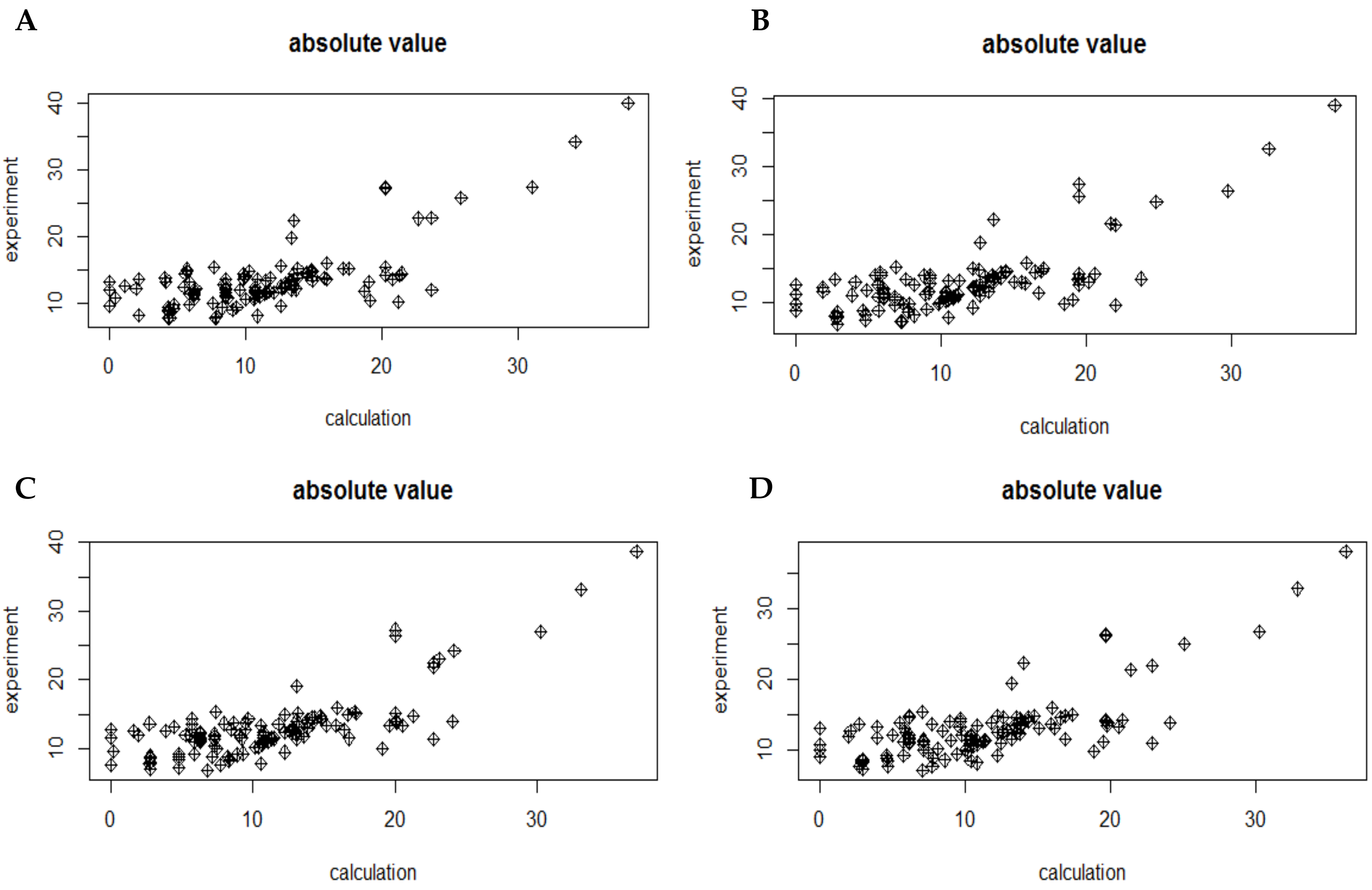

Figure S2: Convergence plots of (A) WT, (B) *pknB*, (C) *stp* and (D) *pknBstp*. The plots show that there is a good correlation between the measured transcriptome data (y-axis) and the flux calculation (x-axis) and we can deduct from this that the flux calculation converged well.

Conservation of *glmR*

As depicted in Figure 6, the *glmR/yvcK* and *pknB* operons are variable between species. Based on our results of pathway analysis and the suggested role of PknB/Stp, we propose that GlmR/YvcK and proteins encoded by the *glmR/yvcK* regulon might play a role in the switch between glycolysis and gluconeogenesis, although the *glmR/yvcK* regulon is variable in different species. As mentioned before, the *glmR/yvcK* regulon seems to have three core genes, which are conserved among the studied organisms. The genes the core comprises are: *yvcJ* (RNase adaptor Rapz), *glmR/yvcK*, and *whiA* (transcription factor). The first one is known to interact with RNase E in *E. coli* to control the translation of some genes. However, RNase E does not have a homologous gene in Firmicutes (*B. subtilis, S. aureus, L. monocytogenes*) (Guillet, Hallier, & Felden, 2013),(Condon, 2003). Thus, YvcJ may interact with other RNases (Even et al., 2005) in order to regulate late competence genes (Luciano, Foulquier, Fantino, Galinier, & Pompeo, 2009). Another significant fact is that YvcJ GTPase function and subcellular localization in *B. subtilis* depend on the growth medium conditions, mainly they are influenced by the presence or absence of glucose (Pompeo, Luciano, Brochier-Armanet, & Galinier, 2011). Moreover, a *yvcJ* homologous gene in *E. coli* is known to regulate the flux through cell wall synthesis (Kalamorz, Reichenbach, März, Rak, & Görke, 2007). The other gene forming the core regulon with *glmR/yvcK* is *whiA*, which is involved in FtsZ-ring assembly in *B. subtilis* and *S. coelicolor* but not proved yet whether it is a transcription regulator (Surdova et al., 2013),(Ainsa et al., 2000). However, the relationship between these three core genes and how they function together remains to be further investigated.

Apart from the core genes, some microorganisms have other different genes inside the *glmR/yvcK* regulon. In *B. subtilis,* there are two uncharacterized enzymes (acetyltransferase and nudix hydrolase) and Crh, a protein required for carbon catabolite repression (Galinier et al., 1997). In *L. monocytogenes* EGD-e, there are several known genes involved in sugar metabolism including galactose, mannose, and fructose for the synthesis of glycoproteins and glycolipids. There are also two short genes located upstream of the core gene, but their functions remain unclear. Lastly, in *M. tuberculosis* the operon includes an extra gene coding for an endoribonuclease that fixes DNA lesions.

The identification of residues involved in UDP-sugar binding in GlmR/YvcK is shown on next page in **Figure S3**, providing the full alignment.

```
                         10        20        30        40        50        60
                          |         |         |         |         |         |
B SUBTILIS      MGHKP------KIAIFGGGTGLSVLLRGLKHKPVXRGLKHKPVDITAIVTVADDGGSSGR
B HALODURANS    MKKK-------NVVVFGGGTGLSVLLR---------GLKTFPVSITAIVTVADDGGSSGR
SA 8325         MRQI-------KVVLIGGGTGLSVMAR---------GLREFPIDITAIVTVADNGGSTGK
SA NEWMAN       MRQI-------KVVLIGGGTGLSVMAR---------GLREFPIDITAIVTVADNGGSTGK
STAPHYLOCOCCI   MRQI-------KVVLIGGGTGLSVMAR---------GLREFPIDITAIVTVADNGGSTGK
SA COL          MRQI-------KVVLIGGGTGLSVMAR---------GLREFPIDITAIVTVADNGGSTGK
S EPIDERMIDIS   MKQM-------NVVLIGGGTGLSVLAR---------GLREFPIDITAIVTVADNGGSTGK
L PLANTARUM     MRKYTFKTQRPKIVVIGGGTGLPVVLN---------GLRKQAVDITAVVTVADDGGSSGI
S PNEUMONIAE    MRK-------PKITVIGGGTGIPVILK---------SLREKDVEIAAIVTVADDGGSSGE
L MONOCYTOGENES MKKET----KPKVVVIGGGTGLPVILK---------GLKKKDVHLTAIVTVADDGGSSGK
M TUBERCULOSIS  MTDG--------IVALGGGHGLYATLS---------AARRLTPYVTAVVTVADDGGSSGR
                * .         :. :*** *: .            . :     ::*:*****:***:*

                         70        80        90       100       110       120
                          |         |         |         |         |         |
B SUBTILIS      LR-NELKIPPPGDIRNVLAALSDVE---PLVEDLFQHRFNKGN-DLTGHSLGNLILAAMT
B HALODURANS    LR-KELDIPPPGDVRNVLVALSEVE---PLLEQLFQHRFENGN-GLSGHSLGNLLLAGMT
SA 8325         IR-DEMDIPAPGDIRNVIAALSDSE---SVLSQLFQYRFEE-N-QISGHSLGNLLIAGMT
SA NEWMAN       IR-DEMDIPAPGDIRNVIAALSDSE---SVLSQLFQYRFEE-N-QISGHSLGNLLIAGMT
STAPHYLOCOCCI   IR-DEMDIPAPGDIRNVIAALSDSE---SVLSQLFQYRFEE-N-QISGHSLGNLLIAGMT
SA COL          IR-DEMDIPAPGDIRNVIAALSDSE---SVLSQLFQYRFEE-N-QISGHSLGNLLIAGMT
S EPIDERMIDIS   IR-DVMDIPAPGDIRNVIAALSDSE---SILTQLFQYRFGE-N-QVDGHSLGNLVIAGMT
L PLANTARUM     IR-NYVNVVPPGDIRNVMVALSSWP---DLYKDIFQYRFQGDDQFFAGHAIGNLIIAALT
S PNEUMONIAE    LRKNMQQLTPPGDLRNVLVAMSDMP---KFYEKVFQYRFSEDAGAFAGHPLGNLIIAGLS
L MONOCYTOGENES IR-EQMDVLPPGDIRNVMLALSNVD---PRVVDLFQYRFAVDG-DLSGHVIGNLILTALS
M TUBERCULOSIS  LR-SELDVVPPGDLRMALAALASDSPHGRLWATILQHRFGGSG-ALAGHPIGNLMLAGLS
                :* .  .: .***:* .: *::.          ::*:**      . ** :***:::.::
                        130       140       150       160       170       180
                          |         |         |         |         |         |
B SUBTILIS      NITGDFFHAVTEMSKVLNVRGKVLPAANASVVLHAEME-------DGRVVSGESTIPEYG
B HALODURANS    SITGDFARGISEMSKVLNVRGKVLPASNRSIILHGEME-------DGTIVTGESSIPKAG
SA 8325         NITNDFGHAIKALSKILNIKGRVIPSTNTSVQLNAVME-------DGEIVFGETNIPKKH
SA NEWMAN       NITNDFGHAIKALSKILNIKGRVIPSTNTSVQLNAVME-------DGEIVFGETNIPKKH
STAPHYLOCOCCI   NITNDFGHAIKALSKILNIKGRVIPSTNTSVQLNAVME-------DGEIVFGETNIPKKH
SA COL          NITNDFGHAIKALSKILNIKGRVIPSTNTSVQLNAVME-------DGEIVFGETNIPKKH
S EPIDERMIDIS   NITNDFGHAIKELSKVLNIKGQVIPSTNASVQLNAVME-------DGEIVHGETNIPKTH
L PLANTARUM     EMKSGVFDAVQELSNMMQVDGHVYPAANEALTLHGKFS-------DGTELVGEAEITAAH
S PNEUMONIAE    EMQGSTYNAMQLLSKFFHTTGKIYPSSDHPLTLHAVFQ-------DGTEVAGESHIVDHR
L MONOCYTOGENES QLNDSYVDAINVLATVLKIRGKVIPATDQPLILNAEME-------DGSIVHGESLIPLQG
M TUBERCULOSIS  EVLADPVAALDELGRILGVKGRVLPMCPVALQIEADVSGLEADPRMFRLIRGQVAIATTP
                .:  .   .:  :. .:   *:: *    .: :.. ..           : *:  *

                        190       200       210       220       230       240
                          |         |         |         |         |         |
B SUBTILIS      QRIKRVFLTPE----QIDPLPETIDVIREADLIIIGPGSLYTSILPNLLVPKIGEEVIKA
B HALODURANS    KKIKRVFLTPK----DTKPLREGLEAIRKADVIVIGPGSLYTSVLPNLLVPGICEAIKQS
SA 8325         KKIDRVFLEPN----DVQPMEEAIDALREADLIVLGPGSLYTSVISNLCVNGISDALIHS
SA NEWMAN       KKIDRVFLEPN----DVQPMEEAIDALREADLIVLGPGSLYTSVISNLCVNGISDALIHS
STAPHYLOCOCCI   KKIDRVFLEPN----DVQPMEEAIDALREADLIVLGPGSLYTSVISNLCVNGISDALIHS
SA COL          KKIDRVFLEPN----DVQPMEEAIDALREADLIVLGPGSLYTSVISNLCVNGISDALIHS
S EPIDERMIDIS   KKIDRVFLEPS----DVEPMNEAIEALEQADLIVLGPGSLYTSVISNLCVKGISEALLRT
L PLANTARUM     KSLERVWVTDK-NGKEPQAVQPVIDAIMAADQIVLGPGSLFTSILPNLTIGNIGRAVCES
S PNEUMONIAE    GIIDNVYVTNALNDDTPLASRRVVQTILESDMIVLGPGSLFTSILPNIVIKEIGRALLET
L MONOCYTOGENES KHINRVYIEPE----NVKPYPTAVEAVKEADLIVIGPGSLYTSILPNLLLTELADEIIAS
M TUBERCULOSIS  GKVRRVRLLPT----DPPATRQAVDAIMAADLVVLGPGSWFTSVIPHVLVPGLAAALRAT
                  : .* :          .    ::.:  :* :::**** :**::.:: :  :   :  :

                        250       260       270       280       290       300
                          |         |         |         |         |         |
B SUBTILIS      PAKKVYICNVMTQPGETLHYTAADHVKALNQHMGCGFIDTILVNSEDIPDEIKR-KYEME
B HALODURANS    TARKVYICNVMTQNGETDGYTASDHLQAIMDHCGVGIVDDILVHGEPISDTVKA-KYAKE
SA 8325         DAPKLYVSNVMTQPGETDGYSVKDHIDAIHRQAGQPFIDYVICSTQTFNAQVLK-KYEEK
SA NEWMAN       DAPKLYVSNVMTQPGETDGYSVKDHIDAIHRQAGQPFIDYVICSTQTFNAQVLK-KYEEK
STAPHYLOCOCCI   DAPKLYVSNVMTQPGETDGYSVKDHIDAIHRQAGQPFIDYVICSTQTFNAQVLK-KYEEK
SA COL          DAPKLYVSNVMTQPGETDGYSVKDHIDAIHRQAGQPFIDYVICSTQTFNAQVLK-KYEKK
S EPIDERMIDIS   SAPKLYVSNVMTQPGETDNYDVKEHIDALTRQVGEPFIDFVICSSESYSKDVLQ-RYEEK
L PLANTARUM     DAEVVYICNIMTQKGETDNFSDADHVRVLNRHLGQNFINTVLVNTEKVPEDYMDFHKFNE
S PNEUMONIAE    KAEIAYVCNIMTQRGETEHFTDSDHVEVLHRHLGRPFIDTVLVNIEKVPQEYMNSNRFDE
L MONOCYTOGENES KAPKVYITNILTQIGETDFFSDADHIKVIHEHVGKSFIDKTLINTTTVPKELL----FPE
M TUBERCULOSIS  SARRALVLNLVAEPGETAGFSVERHLHVLAQHAPGFTVHDIIIDAERVPSERE-------
                 *    : *:::: ***  :    *: .:  :     :.  :

                        310       320       330       340       350       360
                          |         |         |         |         |         |
B SUBTILIS      SARPVDFDIEELK--AMGLEVIRDQIVTYKNDVIRHDTHKVASLLVDLLKE---------
B HALODURANS    KAEPVIVDEHKLK--ALGVGTISDYFVLEQDDVLRHNASKVSEAILEGKPRTSSSIQ---
SA 8325         HSKPVEVNKAELEKESINVKTSSNLVEISENHLVRHNTKVLSTMIYDIALELISTIPFVP
SA NEWMAN       HSKPVEVNKAELEKESINVKTSSNLVEISENHLVRHNTKVLSTMIYDIALELISTIPFVP
STAPHYLOCOCCI   HSKPVEVNKAELEKESINVKTSSNLVEISENHLVRHNTKVLSTMIYDIALELISTIPFVP
SA COL          HSKPVEVNKAELEKESINVKTSSNLVEISENHLVRHNTKVLSTMIYDIALELISTIPFVP
S EPIDERMIDIS   NSKPVAVHKEQLKDSGIRVLTASNLVEISNEHYVRHNTKVLSKMIYELALELTSTIRFTP
L PLANTARUM     VSKQVSHDFRGLR--EQNCRVISSNFLKLRDNGAFHDGDQVVAELMNLVGHSDVFR----
S PNEUMONIAE    YLVQVEHDFVGLC--KQVSRVISSNFLRLENGGAFHDGDLIVDELMRIIQVKK-------
L MONOCYTOGENES DVAQVEHNAKGME--ELGVEAIYQDFLSTEDGLVRHAAEKVADALLAMLPNQTIEKE---
M TUBERCULOSIS  ------------------------------------------------------------
```

Figure S3: Identification of residues involved in UDP-sugar binding in GlmR/YvcK (complete sequence alignment profile of GlmR/YvcK in different bacteria including several S. aureus strains)

Table S14. Flux activities comparing WT and PknB mutant in *S.aureus* strains NewHG and NCTC8325

EPM	WT (NewHG)	PknB (NewHG)	WT (NCTC8325)	PknB (NCTC8325)	Ratio_NewHG	Ratio_NCTC8325
1	13.5274496796	13.0915648782	9.267258786	10.1246456098	0.9677777547	1.0925178463
2	7.7369796456	7.2257999745	9.3491371896	8.4923990841	0.9339303327	0.9083617998
3	13.348656016	12.7122436004	7.6610522535	6.9835599347	0.9523238583	0.9115666756
4	14.8567603522	14.507840714	5.0070897811	4.9370154652	0.9765144197	0.9860049811
5	25.8232973945	24.8555084527	8.9078746257	8.5089068668	0.9625226428	0.9552117901
6	7.867961062	7.3107693647	8.7186813827	7.8709192603	0.9291821994	0.9027648695
7	15.99112194	15.8781628798	15.3464909923	14.768208518	0.9929361391	0.9623182606
8	14.4769752505	14.1136841407	11.6264287919	10.5105343398	0.9749055929	0.9040208759
9	13.5198207211	13.0760480803	13.4777095944	12.6441545207	0.9671761446	0.9381530617
10	23.1328865982	13.6304843704	4.4900038975	7.325498436	0.5892254005	1.6315127121
11	-13.5823568323	5.46374498	5.0070897811	4.9370154652	-0.4022678131	0.9860049811
12	7.2478358552	7.0806790134	5.0070897811	4.9370154652	0.9769369995	0.9860049811
13	34.1926246932	32.593633919	8.4628229969	8.2631610999	0.953235799	0.9764071756
14	7.2478358552	7.0806790134	5.0070897811	4.9370154652	0.9769369995	0.9860049811
15	7.2478358552	7.0806790134	5.0070897811	4.9370154652	0.9769369995	0.9860049811
16	7.2478358552	7.0806790134	5.0070897811	4.9370154652	0.9769369995	0.9860049811
17	11.0054698983	10.508341417	5.9122452766	9.8261955363	0.9548289636	1.662007423
18	13.8240504159	13.679051662	11.0804838672	11.8410830279	0.9895111238	1.068643136
19	22.7048590557	21.6889681778	5.0070897811	4.9370154652	0.9552566754	0.9860049811
20	11.4265098804	11.1972324731	11.772895736	11.1720008305	0.9799346074	0.9489594643
21	11.5562502644	10.9915859489	9.7895477388	8.4046972379	0.9511377564	0.8585378469
22	11.7708198611	10.8318570952	9.2016526615	10.1858818041	0.9202296206	1.1069622142
23	7.2478358552	7.0806790134	5.0070897811	4.9370154652	0.9769369995	0.9860049811
24	12.9579258246	12.2710167669	10.9578837656	10.347035684	0.9469892738	0.9442549223
25	9.0206711284	8.1746761648	5.0070897811	4.9370154652	0.906215962	0.9860049811
26	9.3597519727	9.0476219	10.650807502	9.2592459952	0.9666518863	0.8693468541
27	10.6403453747	9.8548821066	7.8857081044	9.035942933	0.9261806605	1.1458632267
28	12.5657562539	12.208285124	10.3399015841	10.3643007905	0.9715519605	1.0023597136
29	10.8515837552	10.5416164139	8.5072289799	6.7369531983	0.9714357509	0.7919092356
30	0.0473928065	0.0435471597	1.3776462159	0.087540506	0.9188558952	0.0635435317
31	24.6955090397	24.2969232426	19.0930679352	18.204918112	0.9838599886	0.9534831266
32	15.9526644489	15.5476847347	19.5123126132	16.8540239658	0.9746136631	0.8637635272
33	9.8109583069	9.2800341017	2.6943869693	2.9465936518	0.9458845723	1.0936044768
34	13.4885148653	12.729146022	10.2773531338	9.7995695976	0.943702561	0.9535110325
35	10.7119157586	10.1268645264	10.3049040779	11.2408444951	0.9453831373	1.0908247578
36	13.2478966902	12.6690106983	9.1834331057	7.7076882838	0.9563035548	0.8393035802
37	0.6200401003	0.5918240622	0.3947099315	0	0.9544932044	0
39	13.1793476	12.4411135462	10.7012823672	10.7796199928	0.9439855389	1.0073203961
41	4.9337084168	3.7773701474	2.6709179068	3.302636858	0.7656249272	1.2365175468
42	1.2326355616	2.0450977035	3.111887636	2.2608725243	1.6591259958	0.7265276863
43	2.0977259784	2.0835096542	1.3507407487	0	0.9932229832	0
44	2.3124834959	0.4949688036	1.5601179494	0.8250883201	0.2140420913	0.5288627827
45	6.4888720118	6.8196337886	4.7848536183	4.0167344014	1.0509736941	0.839468607
49	11.726350965	11.1837782807	12.0349117486	10.9659888793	0.9537304754	0.911181495
50	14.5633871053	13.5365541612	11.4788240035	10.7103966149	0.9294921616	0.9330569588

51	0.56021387	0.1531324317	0.8100564558	0.8717908683	0.273346377	1.0762100124
52	3.4195918127	3.767004451	0.1870565511	1.3834935355	1.1015947684	7.3961244752
53	0	0.0059603629	1.8833889241	0	0	0
54	0.7094086252	0.4891379403	0.0509939568	0.0327158188	0.6895009772	0.6415626648
55	0.1929500141	0.2643041451	0.3563363075	0.8257867361	1.3698063008	2.3174364179
56	0.2560770205	0.2259853743	0.3561454958	0.3607368947	0.8824898612	1.0128919188
57	1.5954030665	3.6006570016	0.9286045084	1.5165842149	2.2568948733	1.6331863578
58	0.3185108803	0.3653864364	0.0221922091	0.0196243209	1.1471709729	0.884288753
59	0.478152061	0.6600135403	1.4775003711	1.3958673198	1.3803423518	0.9447492177
60	1.2734368797	1.2806982443	0.620940629	0.9215112709	1.0057021787	1.4840569739
61	0.0343693562	0.4662986499	0.3303158938	0.3436285326	13.5672791342	1.04030275
62	0.0824381201	0.4757256629	0.3223622406	0.4331750659	5.7707000423	1.3437524974
63	0.6048830206	1.3870430741	0.3069787047	0.3722353212	2.2930765568	1.2125770142
64	0.2584015597	1.1276185748	0.3016297874	0.3225590568	4.363822634	1.0693872764
65	0.1895292	0.2271635384	0.663348935	0.0666119479	1.1985674949	0.1004176601
66	1.9288397015	1.5404558847	0.8300936936	0.8225336217	0.7986438082	0.9908925077
67	0.6324447894	0.5677134117	0.4434377893	0.5632971903	0.8976489666	1.2702958654
69	1.4735949828	0.1446766307	0.8100764608	0.7065006434	0.0981793725	0.8721406899
70	1.3996537395	0.043500655	0.7994544834	0.6249505159	0.0310795833	0.7817211972
73	15.7959443128	15.0571915101	10.5917649197	10.6574571888	0.9532314885	1.0062022023
74	17.223540549	16.5312309314	15.6409804701	15.5644186316	0.9598044539	0.9951050487
75	17.2116572391	17.0788290015	13.5651508875	14.5924784187	0.9922826584	1.0757328495
76	38.044239692	37.1312416158	6.6014956614	8.2046592808	0.9760016737	1.2428485455
77	8.7167307371	7.8679456276	3.7776452569	7.1645899642	0.902625751	1.8965756383
78	1.3702749117	1.3087798896	1.3644299687	1.4275331416	0.955122128	1.0462487444
79	0.4155011953	0	0.5375644166	0.9054357979	0	1.6843298588
80	0.3632623226	0.2167608827	0.1486301505	0.4630018065	0.5967062072	3.1151270788
81	11.7919359482	11.2775261584	10.8761959355	10.3637568752	0.9563761377	0.9528843482
82	0.2126183491	0.3689632067	0.6504744892	0.2204489585	1.7353309735	0.3389048489
87	4.0833628122	3.8630530285	1.360591683	1.5719824322	0.9460469731	1.1553667804

Flux activity comparison between *S. aureus* NewHG mutant vs WT and NCTC8325 mutant vs WT. As we can observe from the list, the *pknB* mutations on two strains illustrate strain-specific impacts. However, the changes of central metabolism, purine, pyrimidine metabolism, and part of amino acid metabolism remain identical. Results are summarized in the manuscript Table 2.

Table S15: qRT-PCR confirmation of microarray data

Gene product	Gene	Newman ORF	DNA microarray[a] WT/Δ*pknB*	DNA microarray[a] WT/ Δ*stp*	qRT-PCR[b] WT/Δ*pknB* WT/Δ*stp*
Staphopain thiol proteinase	*sspB*	NWMN_ 0917	0.5	5.2	0.6±0.2 5.1±0.8
Serine protease SplB	*splB*	NWMN_ 1705	0.2	8.1	0.8 ±0.3 3.9±0.6
Alpha-hemolysin	*hla*	NWMN_ 1073	0.5	2.4	0.6±0.3 1.5±0.4
Lipase	*lip*	NWMN_ 2569	0.6	1.7	0.5±0.2 2.3±0.5

[a] WT/Δ*pknB* and WT/Δ*stp* mean fold change. The mean ratio ± standard deviation of gene expression level in the mutant strains compared to that of the isogenic wild type strain *S. aureus* NewHG of three independent experiments is shown.

[b] Ratio WT/Δ*pknB* and WT/ Δ*stp*. The mean ratio ± standard deviation of the gene expression level in the mutant strains compared to that of the isogenic wild type strain *S. aureus* NewHG of three independent experiments is shown.
